# Advances in Polysaccharide-Based Microneedle Systems for the Treatment of Ocular Diseases

**DOI:** 10.1007/s40820-024-01477-3

**Published:** 2024-08-13

**Authors:** Qingdong Bao, Xiaoting Zhang, Zhankun Hao, Qinghua Li, Fan Wu, Kaiyuan Wang, Yang Li, Wenlong Li, Hua Gao

**Affiliations:** 1 State Key Laboratory Cultivation Base, Shandong Provincial Key Laboratory of Ophthalmology, Eye Institute of Shandong First Medical University, Qingdao, 266071 People’s Republic of China; 2 State Key Laboratory of Structural Chemistry, Fujian Institute of Research on the Structure of Matter, Chinese Academy of Sciences, Fuzhou, 350002 People’s Republic of China; 3 Eye Hospital of Shandong First Medical University, Jinan, 250021 People’s Republic of China; 4 College of Ophthalmology, Shandong First Medical University, Jinan, 250000 People’s Republic of China; 5 Departments of Diagnostic Radiology, Surgery, Chemical and Biomolecular Engineering, and Biomedical Engineering, Yong Loo Lin School of Medicine and College of Design and Engineering, National University of Singapore, Singapore, 119074 Singapore

**Keywords:** Ocular delivery, Polysaccharide, Microneedles, Drug administration

## Abstract

The eye, a complex organ isolated from the systemic circulation, presents significant drug delivery challenges owing to its protective mechanisms, such as the blood-retinal barrier and corneal impermeability. Conventional drug administration methods often fail to sustain therapeutic levels and may compromise patient safety and compliance. Polysaccharide-based microneedles (PSMNs) have emerged as a transformative solution for ophthalmic drug delivery. However, a comprehensive review of PSMNs in ophthalmology has not been published to date. In this review, we critically examine the synergy between polysaccharide chemistry and microneedle technology for enhancing ocular drug delivery. We provide a thorough analysis of PSMNs, summarizing the design principles, fabrication processes, and challenges addressed during fabrication, including improving patient comfort and compliance. We also describe recent advances and the performance of various PSMNs in both research and clinical scenarios. Finally, we review the current regulatory frameworks and market barriers that are relevant to the clinical and commercial advancement of PSMNs and provide a final perspective on this research area.

## Highlights

- Polysaccharide-based microneedles are novel and emerging tools for ocular drug delivery and the research on the diagnosis and treatment of eye diseases is advancing at a fast pace.
- Microneedle devices constructed from polysaccharide molecules derived from ocular tissue have the potential to significantly enhance the efficiency of clinical treatments and improve patient compliance with therapeutic regimens.
- Guided by our vast clinical experience, this is the first review collates the cutting-edge scientific findings from the interdisciplinary field combining natural macromolecules and ophthalmology.

## Graphical abstract

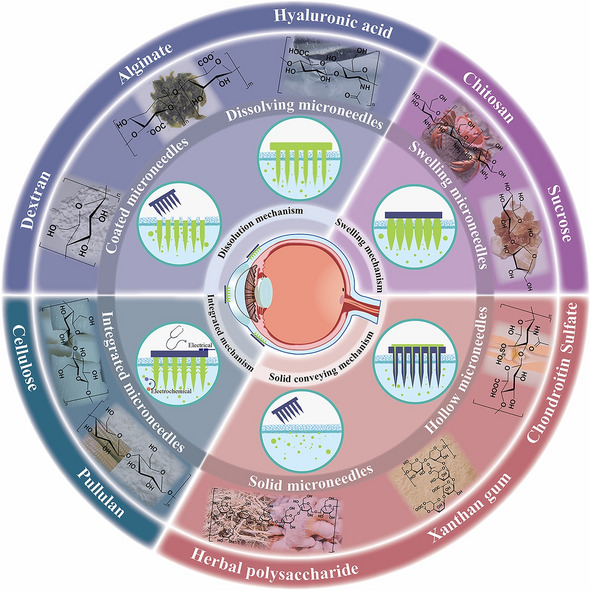

## Introduction

The eye is a complex organ with unique pharmacokinetic and pharmacodynamic characteristics that are segregated from the systemic circulation. The ocular system presents formidable barriers to drug delivery, characterized by protective mechanisms such as the blood-retinal barrier and corneal impermeability. These barriers challenge the efficient administration of therapeutics, often leading to the suboptimal treatment of ocular diseases. Traditional drug administration methods fail to maintain therapeutic levels without causing adverse effects or compromising patient compliance.

Microneedle (MN) technology, which involves an array of micron-scale needles with a diameter of less than 1 mm, has emerged as a promising alternative and is capable of enhancing bioavailability and reducing administration frequency [1, 2]. By penetrating ocular tissues with minimal invasiveness, MNs offer targeted and controlled drug release, which is crucial for the treatment of chronic ocular diseases, such as age-related macular degeneration (AMD) and diabetic retinopathy [3]. However, challenges to its practicality and clinical translation remain unaddressed [4]. Parameters such as the mechanical strength of MNs, insertion depth, and drug-loading capacity require further optimization to meet diverse clinical needs [5]. More importantly, considering the sensitivity of the eye to foreign bodies, traditional MNs may provoke adverse responses, such as increased inflammation, elevated blink frequency, and augmented tear production, which can lead to poor patient compliance [6, 7]. Thus, the development of a gentle, eco-friendly, and safe MN drug delivery platform is imperative to address these concerns and enhance the viability of this promising technology in clinical settings.

Polysaccharides, with their biocompatibility, natural abundance, and modifiable degradation rates, are well-suited for the development of MN systems that can meet the abovementioned requirements [8]. Their versatility and functionality have been exploited in various biomedical applications, including drug delivery systems that prioritize patient comfort [9].

Polysaccharides are vital structural components of the eye that play a crucial role in maintaining its normal physiological state [10]. In the cornea, polysaccharides, such as hyaluronic acid (HA), provide structural support and hydration, maintaining corneal transparency and refractive function. In the sclera, polysaccharides and collagen form a matrix that imparts elasticity and toughness, and protects the eye from external pressure and injury [11]. Within the retina, polysaccharides are involved in cell interactions and signaling processes that significantly affect the survival, differentiation, and synapse formation of retinal neurons [12]. For ocular applications, polysaccharides not only facilitate drug delivery but also offer the potential for formulating devices that minimize discomfort and support patient compliance, thereby enhancing the overall therapeutic outcome [13].

Polysaccharide-based microneedles (PSMNs) significantly affect ophthalmic drug delivery. Their tailored design caters to the specific requirements of ocular diseases and facilitates precise and efficient medication administration, which is a significant advancement in eye disease treatment. PSMNs are an innovative delivery platform that enhances both treatment efficacy and patient adherence, which are crucial in ophthalmology. However, a comprehensive review of PSMNs in this domain has not been published to date.

**Fig. 1:**
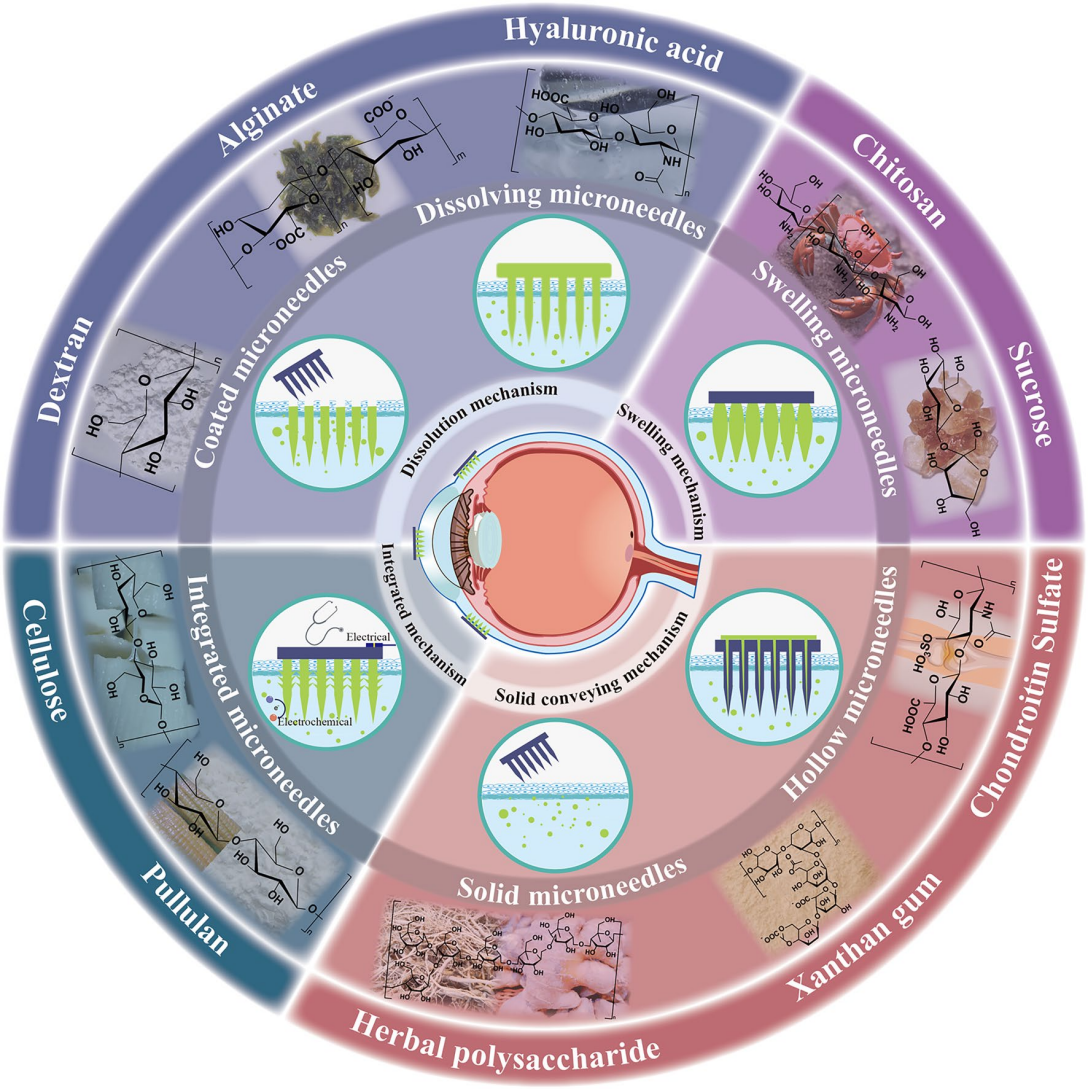
Overview of polysaccharide-based microneedles (PSMNs) technologies and delivery mechanisms for ocular drug administration. *Outer circle* important polysaccharides (hyaluronic acid, chitosan, dextran, alginate, cellulose, chondroitin sulfate, pullulan, and *B. striata* polysaccharide) used in PSMNs. *Middle circle* different types of PSMNs (dissolving microneedles, coated microneedles, swelling microneedles, solid dose microneedles, and integrated microneedles) used for ocular drug administration. *Inner circle* core mechanisms (dissolution, swelling, solid-conveying, and integrated mechanisms) of PSMNs used for ocular drug delivery

To address this issue, this review delves into the intersection of polysaccharide chemistry and MN technology, focusing on ocular drug delivery systems that address treatment efficacy and patient adherence (Fig. 1). With this review, we aim to provide an in-depth analysis of PSMNs and examine their design principles, fabrication techniques, and the challenges that were overcome, including patient comfort and compliance. The latest developments are critically assessed, where both the success and limitations of PSMNs in both preclinical and clinical settings are discussed.

In this review article, the properties of polysaccharides that are crucial for the development of patient-friendly PSMNs are first described. Then, the current fabrication methods for PSMNs and the mechanisms by which these MNs enhance drug delivery while ensuring a comfortable patient experience are presented. Subsequently, the applications of PSMNs in various ocular diseases are reviewed, emphasizing their role in overcoming the limitations of traditional therapeutics. Finally, the regulatory and commercialization aspects of PSMNs that are integral to their translation from bench to bedside are discussed.

## Polysaccharides: Key Regulators in the Ocular Structure

**Fig. 2:**
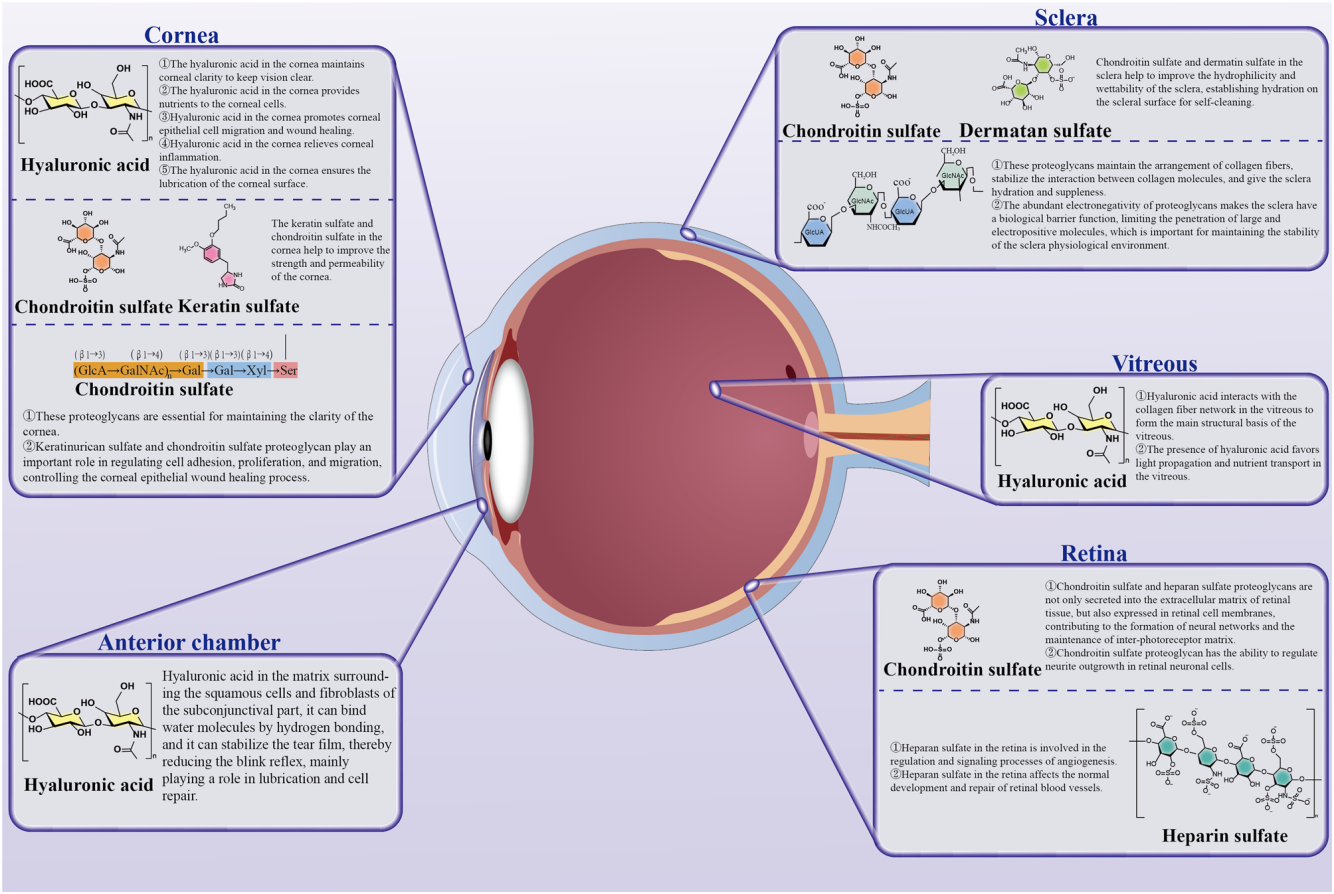
Structural and functional roles of ocular polysaccharides and their critical impact

Polysaccharides are vital biological polymers composed of monosaccharide units linked by glycosidic bonds. Carbohydrates play critical roles in various biological processes and structural functions [14]. Polysaccharides are critical for maintaining the structural and functional integrity of the eye, notably through their role in the composition of the extracellular matrix, ensuring corneal transparency, and regulating intraocular pressure (Fig. 2). The eye is a complex and finely tuned sensory organ, it is anatomically divided into the anterior and posterior segments [15]. The intricate and sophisticated anatomical architecture of the eye is intricately woven with the participation of various polysaccharide molecules, which play crucial roles in the structural integrity and functional aspects of ocular tissues [16, 17].

Within ocular tissues, glycosaminoglycans (GAGs) are the predominant polysaccharides and play a critical role in maintaining structural and functional integrity [18, 19]. These linear polymers, characterized by repeating disaccharide units of amino sugars and uronic acids, exhibit molecular weights of 10–100 kDa and pervade the extracellular matrix and collagen fiber interstices. The isolation and subsequent characterization of GAGs, initiated in the late nineteenth century by Mörner et al. led to the discovery of various ocular GAGs, including HA, chondroitin sulfate, and heparin (HP) sulfate [20, 21].

HA, first extracted from bovine vitreous bodies by Meyer et al. [22] in 1934, is integral to the vitreous body structure, enhancing light transmission and nutrient diffusion. Its interaction with the collagen network is fundamental to the viscoelastic properties that are vital for ocular function. Despite its lower concentration in the limbal region than in the vitreous body, HA is critical for limbal stem cell as it supports corneal transparency, nutrient delivery, cellular migration, and wound-healing processes. Moreover, HA contributes to the maintenance of corneal surface moisture and the mitigation of inflammation. Its presence in the conjunctiva and aqueous humor also plays a significant role in lubrication and cellular repair, which involve stabilizing the tear film and the blink reflex frequency through its hydrophilic interactions [23].

In addition to HA, a diverse range of GAGs exist primarily as sulfated derivatives: chondroitin sulfate, dermatan sulfate (DS), keratan sulfate (KS), and HP. The introduction of sulfate groups into GAGs leads to conformational changes, including chain bending and elongation, which alter their spatial structure and enhance their electrostatic repulsion. These molecular transformations increase aqueous solubility and bioactivity, facilitating various biological processes such as immunomodulation, antiviral defense, and antioxidative protection [24, 25]. The enhanced properties of sulfated GAGs are crucial for reinforcing corneal integrity, contributing to the structural and functional maintenance of the retinal vasculature, and improving the hydrophilic and wetting characteristics of the sclera, which are key factors in ocular health and the development of biomaterials for vision restoration therapies [26, 27].

The anionic properties of sulfated GAGs facilitate their covalent bonding with core proteins to form proteoglycans [28]. Through specialized domains on their GAG side chains, these complex molecules engage in targeted interactions with numerous bioactive molecules, demonstrating their multifunctionality. Proteoglycans are categorized based on their GAG chains into chondroitin sulfate, DS, heparan sulfate, and KS proteoglycans, each of which contributes distinctively to ocular tissue structure and function [29]. In the corneal stroma, proteoglycans are critical for clarity and transparency, and alterations in their levels can lead to visual impairment due to corneal haziness. In the retina, chondroitin sulfate and heparan sulfate proteoglycans are central to synaptic connections within neural networks and the structural integrity of the matrix surrounding photoreceptors. However, the overexpression of these molecules can result in pathological states such as retinal neurodegeneration [20].

Moreover, proteoglycans such as chondroitin sulfate can regulate the growth of neurites in retinal neurons, where excess levels may inhibit the repair and regeneration of damaged neuronal pathways [30]. In the sclera, proteoglycans constitute a significant portion of the soluble matrix, despite comprising less than 1% of the dry weight of the matrix. Key scleral proteoglycans, including decorin, biglycan, aggrecan, and lumican, contribute to tissue hydration and flexibility by organizing the collagen fibrils and modulating intermolecular forces [31]. Their negative charge also creates a barrier against the infiltration of large and positively charged entities, thereby maintaining scleral homeostasis [32]. However, disruptions in the enzymatic processes that regulate proteoglycan levels can lead to excessive accumulation of negatively charged molecules, inducing tissue edema and ocular discomfort and potentially exacerbating conditions such as glaucoma or scleritis [33, 34].

Therefore, PSMN drug delivery systems offer significant advantages for ocular therapeutics, namely enhanced biocompatibility, precise bioactive molecule release profiles, and minimal invasiveness, which are crucial considering the delicate nature of ocular tissues. Such systems can bridge the gap in the current treatment modalities by offering a targeted and patient-friendly approach to improving clinical outcomes. The potential of PSMNs for clinical applications is underscored by promising preclinical data, which suggests a paradigm shift in the management of ocular diseases.

## Polysaccharides in PSMNs

The heterogeneity of polysaccharides provides a versatile molecular basis for optimizing the properties of PSMNs. They are integral to biological structures and functions, as they provide essential support, energy storage, lubrication, and participation in cell signaling [35]. Hence, their high biocompatibility made them prime candidates for creating natural PSMN matrices for therapeutic delivery and diagnostic applications.

Polysaccharides can be economically sourced from natural reserves including algae (alginates), plants (starch, cellulose, and pectin), animals (chitosan (CS), HA, and chondroitin), and microbes (dextran, xanthan, pullulan) [36]. The malleability of their physical, chemical, and biological properties facilitates versatile modifications that enhance the efficacy and safety profiles of PSMNs [37]. PSMNs containing HA, CS, cellulose, trehalose, dextran, and herbal polysaccharides demonstrated significant potential in applications [38, 39]. These polysaccharides are used in their pure form, as part of blends, or within composite materials to exploit their unique properties, such as molecular weight and swelling behavior, which profoundly influence drug release dynamics [40] (Table 1).

**Table 1:** Comparative table of polysaccharides types

Material type	Source	Features	Application examples
Hyaluronic acid (HA)	Animal tissues	Excellent viscoelasticity, biocompatibility, and biodegradability	Treatment of ocular diseases, e.g., corneal neovascularization
Chitosan (CS)	Crustaceans	Cationic property, antimicrobial, biodegradable	Treatment of ocular infections, e.g., bacterial keratitis
Dextran	Microorganisms, plants	Good aqueous solubility, biocompatibility, and hydrogel-forming ability	Drug delivery and aesthetic treatments
Alginate	Algae, bacteria	Gelation ability, protein stabilizing for vaccine delivery	Transdermal delivery of therapeutic agents
Starch and pullulan	Plants, microorganisms	Biodegradable, processability, and film-forming capabilities	Insulin release systems, enhanced transdermal absorption
Cellulose	Plants	Abundant source, water insolubility at ambient temperatures	Cancer therapy, Alzheimer’s disease management
Chondroitin sulfate	Animal connective tissue	Sulfated GAG, improved bioavailability	Vaccine delivery, immunization
Other polysaccharides	Various sources	Diverse physical and chemical properties depending on the specific polysaccharide	Protein stabilizers, thickeners, etc

### Hyaluronic Acid

**Fig. 3:**
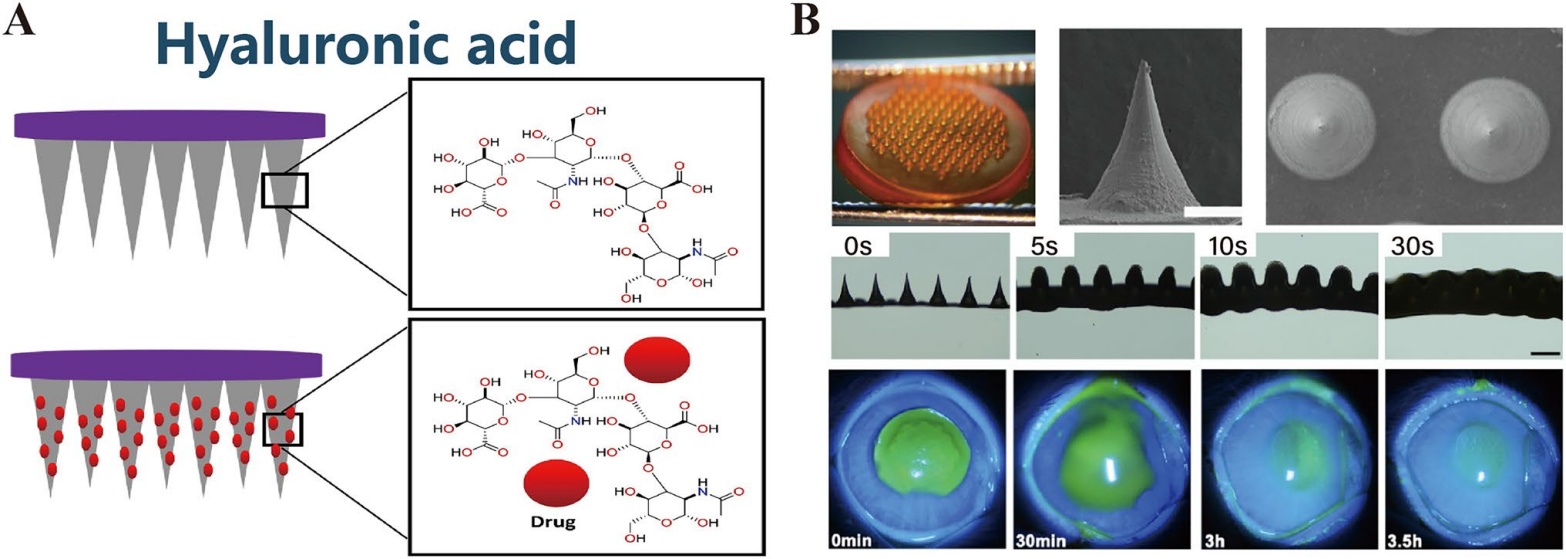
**A** Hyaluronic acid. Reproduced with Permission [42]. Copyright 2021, Elsevier Ltd. **B** Biodegradable composite microneedle patch engineered for the targeted and efficient application of curcumin to the ocular surface, incorporating a fusion of methoxy-poly(ethylene glycol)-poly(ε-caprolactone) and hyaluronic acid as the foundational matrix. Reproduced with Permission [44]. Copyright 2023, Elsevier B.V

HA is a ubiquitous linear GAG with repetitive disaccharide units of *N*-acetyl-d-glucosamine and d-glucuronic acid, with molecular weights of 1–10,000 kDa (Fig. 3A) [41, 42]. It is abundant in the vitreous body of the ocular cavity and demonstrates optimal viscoelasticity, inherent transparency, biocompatibility, and biodegradability [13]. Therefore, HA is an ideal candidate for ocular drug delivery systems such as PSMNs to enhance therapeutic efficacy [43].

HA MNs have inherent hydrophilicity, which has been exploited in the development of dissolving microneedles (DMNs) [45, 46]. Shi et al. [44] used a simple micromolding process to fabricate HA MN patches loaded with curcumin. In a rabbit model of endotoxin-induced uveitis (EIU), these patches exhibited a significantly higher therapeutic effect than that of curcumin eye drops (Fig. 3B) [44]. However, the rapid dissolution of HA in aqueous solutions poses a significant challenge. Wu et al. [47] demonstrated that HA MNs with molecular weights of 200–400 kDa were deformed under a compressive force of 3 N/array, losing more than 30% of their height. These MNs dissolved within 30 s, and the incorporation of ovalbumin (OVA) as a model protein extended the dissolution time to 90 s. Moreover, ex vivo permeation studies indicated that OVA-loaded HA MNs did not significantly enhance permeation (38.43 ± 6.38%).

Further exploration of the performance of low-concentration polysaccharide materials in MNs was conducted by Bonfante et al. [48] who examined carboxymethyl cellulose (CMC), alginate, and HA. They assessed various parameters including geometric dimensions, puncturing ability, and dissolution time. Their findings revealed that MN arrays formulated with 3% (w/w) concentrations of these materials dissolved rapidly within 2 min, with 3% HA arrays exhibiting the fastest dissolution at 60 s and 30 s. Importantly, such rapid dissolution rates (< 1 min) could lead to the dissolution of MNs before insertion into ocular tissues, which results in drug deposition on the tissue surface rather than intra-tissue deliver, thereby drastically diminishing its bioavailability.

To ameliorate this rapid dissolution and facilitate controlled and sustained drug release, specifically for ocular therapies, researchers have focused on the chemical modification of HA to enable effective crosslinking. Strategic modifications were performed to modulate the drug release rate [49]. Notably, Than et al. [50] used micromolding to construct a bilayer configuration within MNs to orchestrate a tiered drug release that effectively inhibits corneal neovascularization. These bilayer MNs feature an outer layer of methacrylated hyaluronic acid (MeHA), which provides structural integrity and controls the release profile, whereas the interior core is packed with unmodified HA, which rapidly dissolves and initiates delivery. This dual-layered design ensured that the HA core, impregnated with an anti-inflammatory agent (diclofenac), dissolved expediently to deliver an initial burst of medication. This was followed by the controlled release of an anti-angiogenic monoclonal antibody (DC101) from the surrounding MeHA matrix. Empirical data confirmed that the crosslinked MeHA matrix sustained drug release over 5–6 days. Furthermore, the integration of HA-IgG conjugates into this matrix extended drug release to up to 2 weeks, which confirms the therapeutic efficacy of HA MNs in drug delivery applications.

Moreover, innovative approaches to ocular pharmacotherapy have focused on optimizing the mechanical robustness and drug release profiles of HA MNs. Researchers have incorporated alternative polysaccharides, such as trehalose, into HA MNs and merged HA with biodegradable and biocompatible polymers [51]. These strategies not only enhance the rigidity of HA MNs but also enable sustained drug delivery to ocular tissues, which is a pivotal advancement in therapeutic interventions [52, 53]. In addition, poly(lactic-*co*-glycolic acid) (PLGA) has already been used in U.S. Food and Drug Administration (FDA)-approved intraocular implants for extended drug release [54]. Suriyaamporn et al. [55] have also incorporated poly(methyl vinyl ether-alt-maleic acid) (Gantrez® S-97, or GAN) into HA MNs. This approach facilitates the targeted delivery of fluorescein sodium (FS) to the sclera and offers a novel treatment modality for dry eye syndrome. The GAN–HA MNs demonstrated facile scleral insertion, necessitating a force of merely 0.08 N per needle and achieving a penetration depth of 329.63 µm. Remarkably, these MNs exhibit an in vitro permeation efficiency of 52.15 ± 20.59%, comprising 20.06% GAN, 5% HA, and 1% FS. This underscores the ability of MNs to overcome the scleral barrier, which suggests enhanced drug delivery efficacy.

Further investigations indicate that augmenting HA MNs with polylactic acid (PLA) can significantly increase their hardness (and, consequently, mechanical strength) and facilitate corneal penetration [56]. The addition of methoxy poly(ethylene glycol)-poly(ε-caprolactone) (MPEG-PCL) further refined the mechanical properties and dissolution rates of HA MNs, providing a customizable platform for ocular drug delivery [44]. The molecular architecture of these polymers, comprising polar and nonpolar segments, confers chemical versatility to HA and enables effective interactions with diverse chemical entities. This property renders HA a suitable carrier for drug conjugation, enhancing both the penetration efficiency of MNs and the bioavailability of therapeutic agents [57, 58]. To illustrate this capability, Shi et al. [59] have conjugated a cell-penetrating tumor-targeting peptide, tLyp1, with amphiphilic HA (HA-GMS). They attached two chemotherapeutic agents, doxorubicin (DOX, an IDC inducer) and 1-methyl-d-tryptophan (1MT, an IDO inhibitor), to MNs, achieving improved targeted delivery and a consequent reduction in tumor size [60]. Therefore, the selection or amalgamation of polymers at various concentrations and types facilitates the customization of mechanical and release properties of HA MNs, with release durations ranging from days to months. The strategic incorporation of composite materials into MNs exploits the rich potential of HA as a natural polysaccharide in drug delivery systems, thereby harnessing the full scope of biomaterial adaptability for specialized applications [61].

Moreover, hyaluronic acid, a highly biocompatible and biodegradable polysaccharide, has demonstrated multifaceted advantages in microneedle technology. It serves not only as a drug carrier, enhancing the stability and bioavailability of pharmaceuticals, but also, when integrated with microneedles, augments drug penetration, enabling more effective delivery to challenging tissues such as the eye. Furthermore, hyaluronic acid-based microneedles have exhibited significant therapeutic efficacy in antimicrobial and anti-inflammatory treatments, rapidly eliminating pathogens and mitigating inflammatory responses, thus promoting wound healing. These findings suggest that hyaluronic acid-based microneedles offer an efficacious drug delivery platform for ocular diseases and other conditions requiring localized treatment, holding substantial potential for clinical application. The integration of hyaluronic acid into microneedle formulations is poised to revolutionize targeted drug delivery, with promising prospects for advancing patient care in various therapeutic areas. For instance, Liu et al. [62] have developed an innovative ocular microneedle system, integrating a hyaluronic acid and polymethyl methacrylate (PMMA) matrix to encapsulate multifunctional nanozymes (MnO_x_/GDY) for the treatment of bacterial and fungal keratitis. This hyaluronic acid-based microneedle (MGMN) system demonstrates not only antimicrobial and anti-inflammatory properties but also the capacity to penetrate ocular barriers, thereby enhancing bioavailability. Compared to commercially available ocular antifungal medications, the MGMN system exhibits superior therapeutic efficacy. Furthermore, Liang and colleagues have explored an ultrasound-activated microneedle system that integrates hyaluronic acid with titanium dioxide (TiO_2_) nanosheets for enhanced sonochemical and sonothermodynamic antimicrobial therapies [63]. In this context, the application of hyaluronic acid in microneedles has improved the stability and biocompatibility of the therapeutic agents, while the ultrasound activation of the microneedles has significantly amplified the antimicrobial efficacy, achieving an elimination rate exceeding 99.9999% against multidrug-resistant (MDR) pathogens. Building upon this, Shi et al. [64] have designed a multifunctional heterostructure composed of ultra-small platinum-ruthenium nanoalloys and porous graphitic carbon nitride (C_3_N_4_) nanosheets, integrated within hyaluronic acid microneedles for combined antibacterial and anti-inflammatory therapies. These hyaluronic acid-based microneedles have demonstrated nearly 100% broad-spectrum antibacterial effects against various bacterial strains both in vitro and in vivo, and have effectively suppressed inflammatory responses and promoted wound healing in mice with bacterial infections after just 1 h of visible light irradiation.

In conclusion, HA, a naturally derived polysaccharide, has emerged as a prominent microneedle material for ocular applications due to its inherent properties. Its ubiquity in the vitreous body of the eye is indicative of its exceptional biocompatibility. The viscoelastic nature, optical clarity, and tunable degradation profile of hyaluronic acid, coupled with its capacity for drug loading, make it an ideal candidate for microneedle formulations targeting the ocular region. These intrinsic characteristics not only minimize ocular irritation and enhance patient comfort but also facilitate the effective delivery of therapeutic agents to the eye. Consequently, hyaluronic acid stands out as a preferred material for the development of ocular microneedles.

### Chitosan

**Fig. 4:**
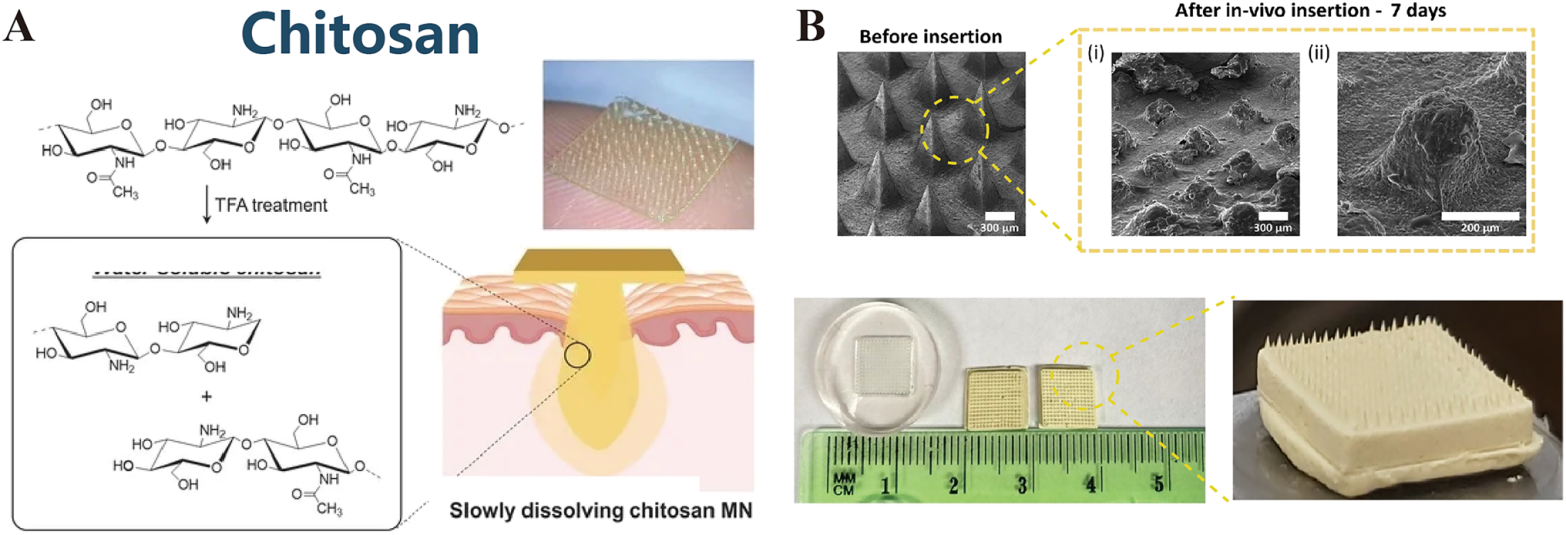
**A** Chitosan. Reproduced under the terms and conditions of the Creative Commons Attribution (CC BY) license [67]. Copyright 2019, The Authors, published by MDPI. **B** Chitosan-based microneedle array patch designed for controlled drug release for different treatments. Reproduced under the terms of the Creative Commons Attribution License [69]. Copyright 2022, Castilla-Casadiego et al.

CS, a polysaccharide with typical molecular weights of 300–1000 kDa depending on its source, has garnered considerable interest for the development of drug delivery systems, particularly in the realm of ocular drug delivery (Fig. 4A) [65–67]. As outlined in a comprehensive review by Yang et al. [68] CS exhibits superior biocompatibility and biodegradability, and its self-assembly capabilities offer a novel strategy for drug delivery. This self-assembly process, which requires no complex manipulation, can form nanostructures with specific functions and properties, a feature of paramount importance for ocular drug delivery given the sensitive and impermeable nature of the ocular environment. CS-based microneedles, as an emerging drug delivery modality, have the potential to enhance bioavailability and therapeutic efficacy. The self-assembled chitosan microneedles enable controlled drug release and targeted delivery, penetrating ocular barriers to deliver medications directly to ocular tissues, thereby reducing systemic side effects and improving therapeutic outcomes. Moreover, the self-assembling nature of CS microneedles confers a high degree of adaptability and flexibility in ocular drug delivery. By adjusting the molecular weight of chitosan, its degree of deacetylation, and incorporating various hydrophilic or hydrophobic side chains, the physicochemical properties of the microneedles can be optimized to cater to different drugs and therapeutic requirements. Despite the immense potential of CS microneedle technology in ocular drug delivery, current research is predominantly focused on theoretical exploration and laboratory studies. Translation to clinical application necessitates overcoming challenges such as FDA approval, in vivo studies, toxicity assessments, biodistribution, and metabolism research. With ongoing research and technological refinement, chitosan microneedles are poised to become an effective therapeutic tool for the treatment of ocular diseases, offering patients safer and more efficacious treatment options.

**Fig. 5:**
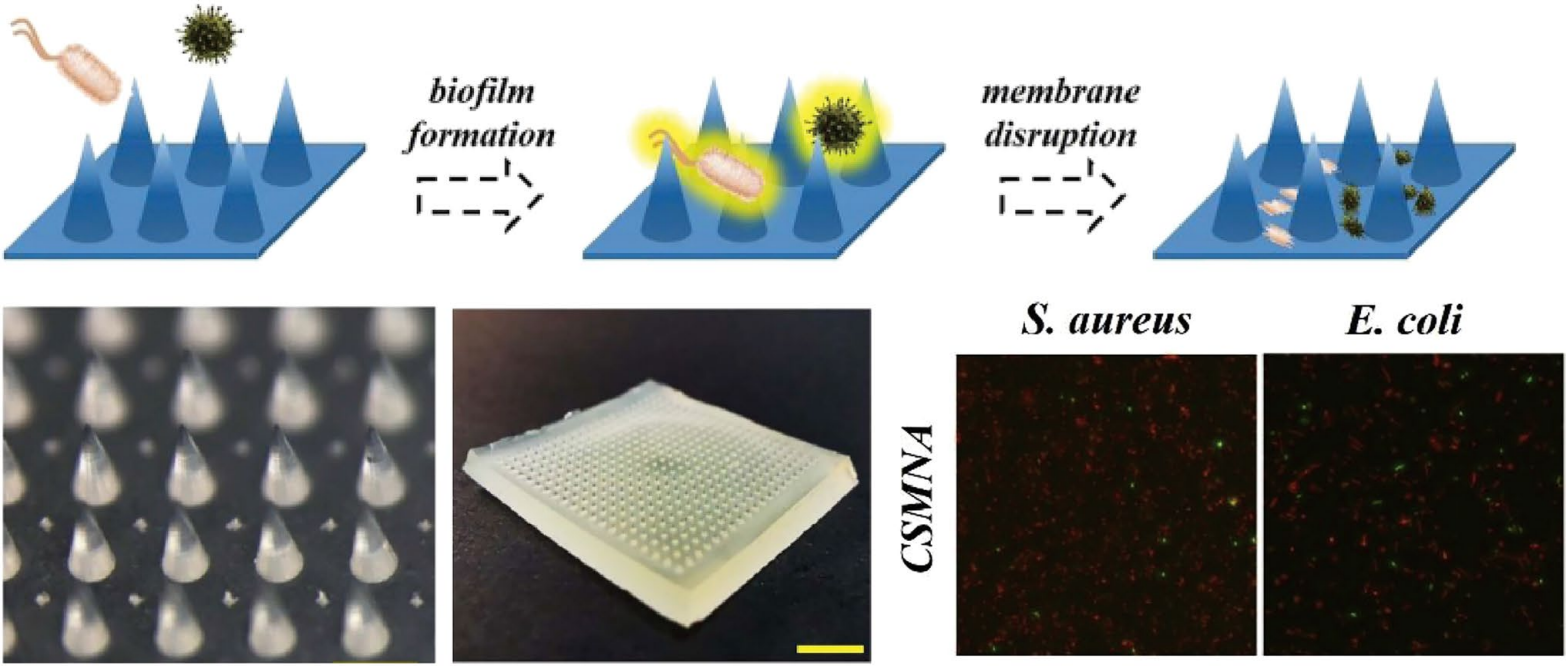
Chitosan-based microneedle array patch designed for controlled drug release for antibacterial treatments. Reproduced under the CC BY-NC-ND license [80]. Copyright 2020, publishing services by Elsevier B.V. on behalf of KeAi Communications Co., Ltd

CS degrades through depolymerization, enzymatic or non-enzymatic hydrolysis, and oxidation, where the degradation rates are influenced by factors such as molecular size and distribution, degree of deacetylation, and moisture content. The cationic character of CS is pivotal for its interaction with anionic cell surfaces, facilitating the opening of tight junction proteins and enhancing drug permeability, making it a valuable candidate for a drug delivery vehicle [70]. Chen et al. [71] have reported CS MN patches that enable sustained delivery of hydrophilic drugs, achieving 95% in vitro drug release within 8 days and demonstrating diffusion of the incorporated BSA molecules to a depth of 300 μm. Ryall et al. [72] delivered asiatic acid (AA) using CS MN arrays, with a sustained release of 52.2% over 48 h. Castilla-Casadiego et al. [69, 73] used CS MNs for the delivery of meloxicam, with a steady release of 33.02 ± 3.88% over 7 d and a dissolution percentage of > 50% of the original MN height after this time frame (Fig. 4B). CS is a naturally occurring bioactive macromolecule and exhibits anti-inflammatory and antimicrobial properties, which make it suitable for the treatment of ocular infections [74–77]. CS MNs have been previously used for wound healing and ocular inflammatory diseases [78, 79]. Chi et al. [80] combined the thermosensitive hydrogel poly(*N*-isopropylacrylamide) (pNIPAM) with CS to prepare multifunctional CS MNs (Fig. 5). Suriyaamporn et al. [74] developed novel bilayer DMNs loaded with fluconazole (FLUZ) microemulsions (MEs) that exhibited high antifungal efficacy in corneal tissues infected with *Candida albicans*. The optimal DMN formulation that exhibited optimal penetration depth and dissolution time on porcine eye tissues comprised 3% CS and 20% PVA. These MNs also showed a mechanical resistance of 40.14 ± 2.10 N and a higher insertion force into corneal tissue (1.78 N/array) than into scleral tissue (0.95 N/array). Additionally, electrostatic interactions between the positive charge of CS and the negative charge on tissue surfaces facilitate mucosal adhesion, making CS-based MNs suitable for targeted ocular diseases [81].

However, the solubility of CS, particularly at the pH level of the ocular environment, limits the applications of CS MNs [82]. To improve CS solubility, Chandrasekharan et al. processed CS with trifluoroacetic acid, inducing random cleavage of glycosidic bonds within the CS chains, followed by simple dialysis in 0.1 M NaCl to yield a water-soluble form of CS. Moreover, CS derivatives with enhanced water solubility were obtained by grafting hydrophilic groups onto the abundant hydroxyl and amine groups present in each CS unit. These derivatives include carboxymethyl chitosan (CMCS), *N*-acetylated chitosan, sulfated chitosan, aminoethyl chitosan, 2-hydroxypropyltrimethyl ammonium chloride chitosan, quaternized chitosan, and thiolated chitosan, which facilitate efficient drug delivery [83, 84]. Wei et al. [85] grafted sericin proteins onto CMCS molecules to create a novel CMCS derivative that was crosslinked with oxidized pullulan (OPL) to form CMCS-based hydrogel MNs. These hydrogel MNs achieved drug loading and efficiency rates of 34.31% and 68.53%, respectively, demonstrating significantly high transdermal delivery of water-soluble Danshen extracts and offering new possibilities for the delivery of water-soluble medications.

### Dextran

**Fig. 6:**
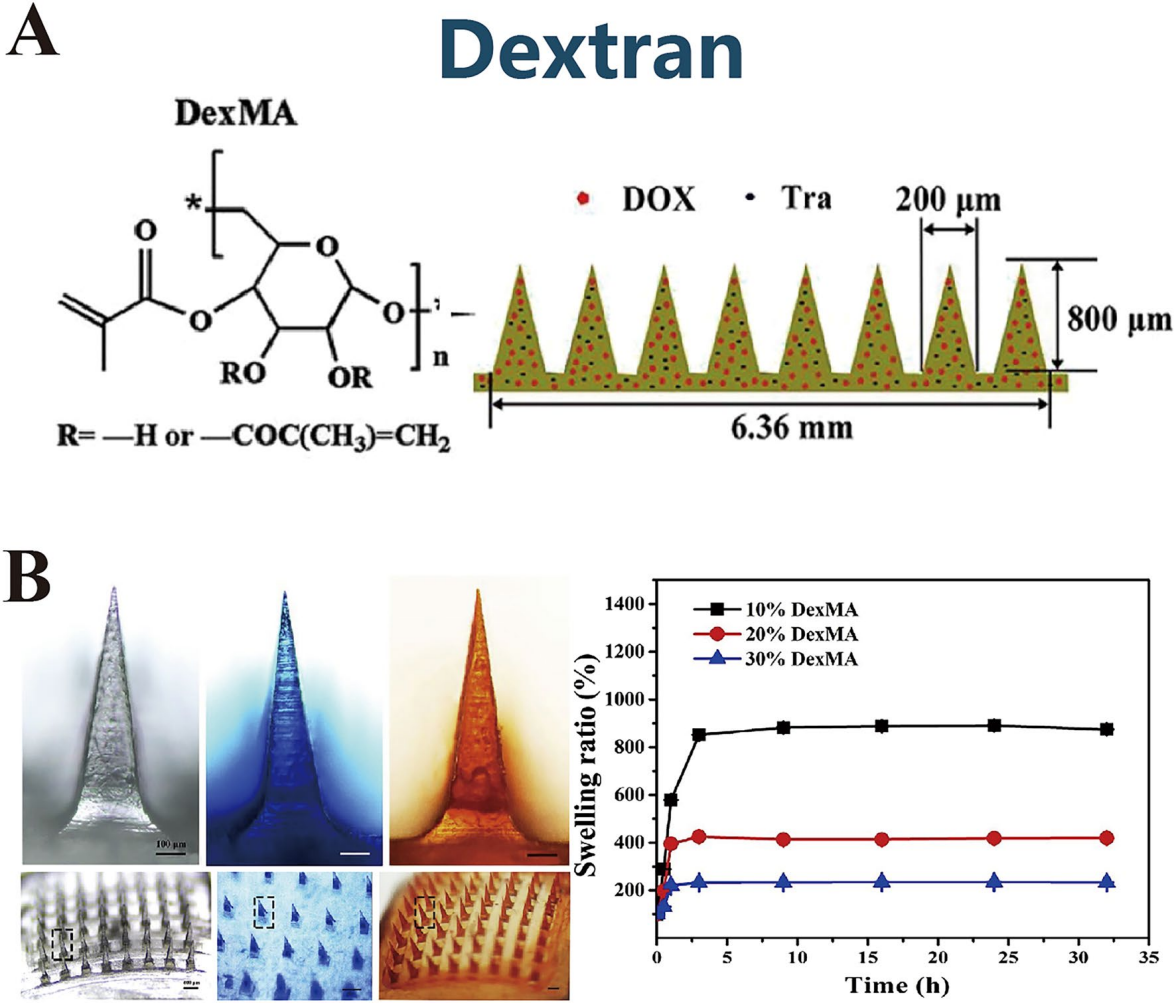
**A** Dextran. Reproduced with Permission [87]. Copyright 2020, Elsevier Ltd. **B** Photopolymerizable dextran methacrylate system for sustained transdermal drug delivery. Reproduced with Permission [87]. Copyright 2020, Elsevier Ltd

Dextran is a branched polysaccharide composed of glucose monomers linked primarily through α-1,6 glycosidic bonds and, to a lesser extent, α-1,3 linkages. Its most physiologically active variant, β-dextran, exhibits immunostimulatory properties and is naturally present in the cell walls of fungi and plants [86]. Dextran has excellent aqueous solubility, biocompatibility, biodegradability, and the ability to form hydrogels. Dextran-based MNs have been widely adopted for drug delivery and aesthetic treatments (Fig. 6A) [87]. Liang et al. [88] fabricated a robust dual-crosslinked hydrogel MNs system using dextran methacrylate (DexMA) combined with hydroxypropyl-*β*-cyclodextrin. This system facilitated the targeted delivery of tofacitinib and a melanocyte-stimulating hormone (α-MSH) to areas affected by vitiligo in mice and achieved a marked increase in melanin deposition after 4 weeks. Huang et al. [87] employed photocrosslinked DexMA to engineer hydrogel MNs (Fig. 6B) that exhibited a high capacity for crosslinking and a significant swelling ratio, which is favorable for sustained drug delivery. Moreover, the inherent stabilizing properties of dextran, particularly under thermal stress, make it suitable for use as a stabilizer in biologics, facilitating the development of thermally stable DMNs [89]. Subsequently, Leelawattanachai et al. [90] formulated dextran MNs for AA delivery and Liu et al. [91] demonstrated its application in stabilizing therapeutic peptides for diabetes treatment. Their comparative study of the storage stability of exenatide (EXT) encapsulated in dissolving MNs composed of various polymers at 50 °C identified dextran as an optimal stabilizer. Dextran and polyvinyl alcohol retained 82.1 ± 3.2% and 76.4 ± 1.2% of dextran, respectively, after 9 weeks of storage at elevated temperatures, which highlights the exceptional stability afforded by the dextran MNs.

The potential of dextran extends to ophthalmic applications, such as eye drop formulations, to mitigate ocular discomfort. Bernd et al. [92] showed that the intraocular distribution of dextran after injection in murine models depends on the molecular weight of the polysaccharide. Nevertheless, the exploration of dextran MNs in ophthalmic research is in its infancy and, hence, presents a substantial opportunity for future investigation.

### Alginate

**Fig. 7:**
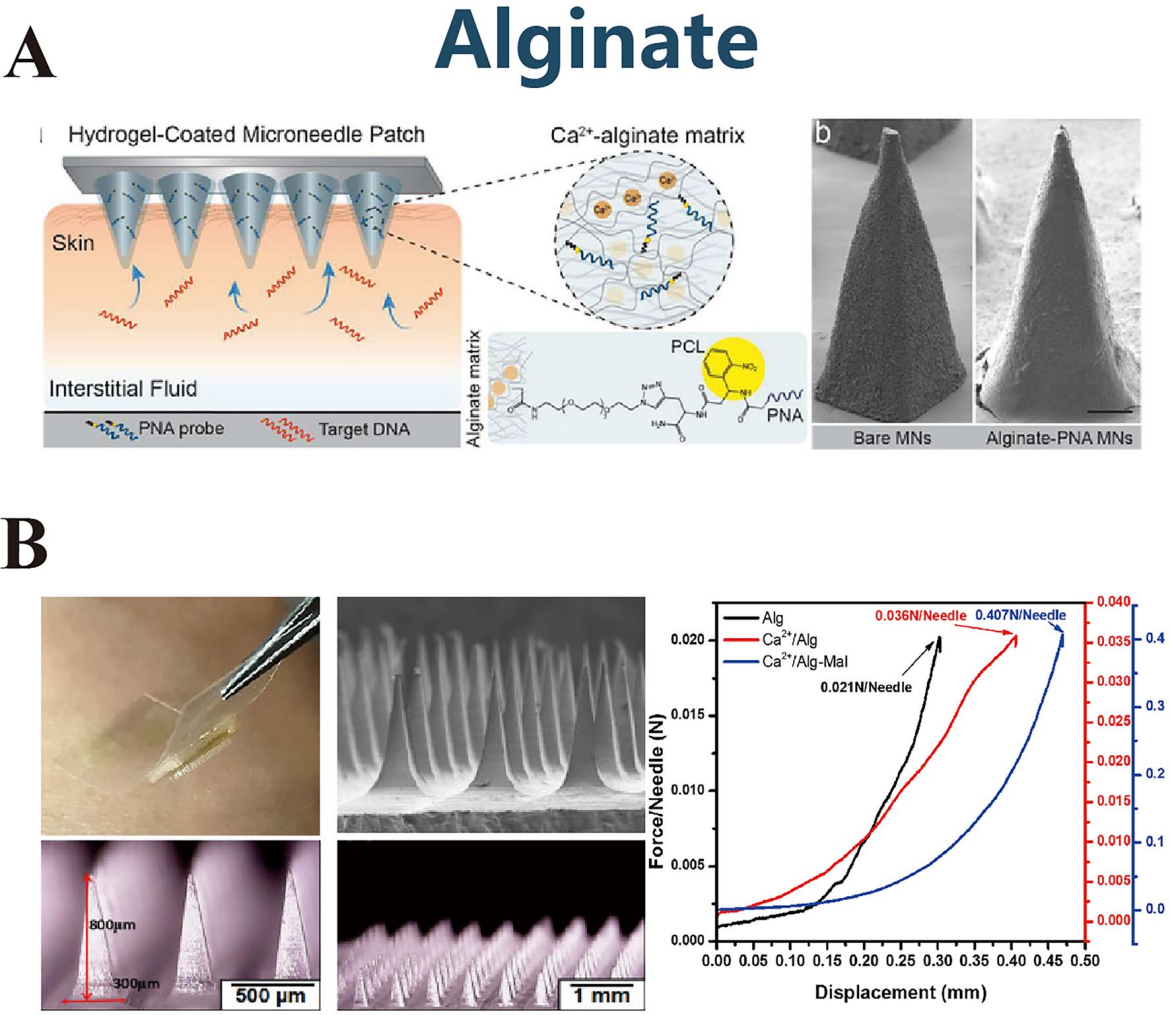
**A** Alginate. Reproduced with Permission [95]. Copyright 2019, American Chemical Society. **B** Alginate and maltose-based microneedle system, created for the non-invasive transdermal administration of insulin. Reproduced with Permission [98]. Copyright 2017, Elsevier B.V

Alginate, an anionic linear polymer derived from algae and bacteria, contains varying ratios of β-d-mannuronic acid (M) and α-l-guluronic acid (G) residues [93]. MNs based on alginate and its derivatives (Fig. 7A) have been used as protein stabilizers for vaccine delivery [94, 95]. Liew et al. [96] assessed the influence of 17 sodium alginate variants on drug release kinetics and concluded that the particle size, viscosity, and concentration of the alginate dictate the release rate and modulate the release mechanism. The gelation capability of alginate, facilitated by crosslinking with divalent metal ions (typically calcium), imparts unique properties conducive to drug release that are instrumental for MN fabrication [97].

Historically, alginate MNs have been used for the transdermal delivery of therapeutic agents, such as bovine serum albumin [99]. Recent advancements include the development of composite MNs that synergistically combine alginate with other polysaccharides to optimize delivery and mechanical properties. Zhang et al. [98] engineered Ca/Alg-Maltose MNs (Fig. 7B), leveraging the combined mechanical resilience of alginate and maltose, to effectively administer insulin. Tiraton et al. [100] developed sodium alginate–gelatin (S–G) MNs and verified their potential in treating acne. Zhou et al. [101] refined the crosslinking process of alginate MNs and tailored them for the encapsulation of acidic drugs. In a novel approach, Yu et al. [102] chemically modified alginate with 3-aminophenylboronic acid to yield Alg-APBA, which formed glucose-responsive complexes. By chemically cross-linking Alg-APBA with HA, they encapsulated insulin within the MNs to achieve a self-regulating insulin release system. This intricate design not only exemplifies the versatility of alginate in drug delivery systems but also shows its potential for responsive therapeutics.

### Starch and Pullulan

Starch, a naturally abundant biodegradable polymer, is widely used in biomedicine owing to its processability and film-forming capabilities [103]. Zhang et al. [104] developed a glucose-responsive insulin release system employing DMN patches. To improve the mechanical strength of these gelatin-starch-based MNs, nanomaterials were incorporated to achieve a resistance of 0.29 N per needle.

**Fig. 8:**
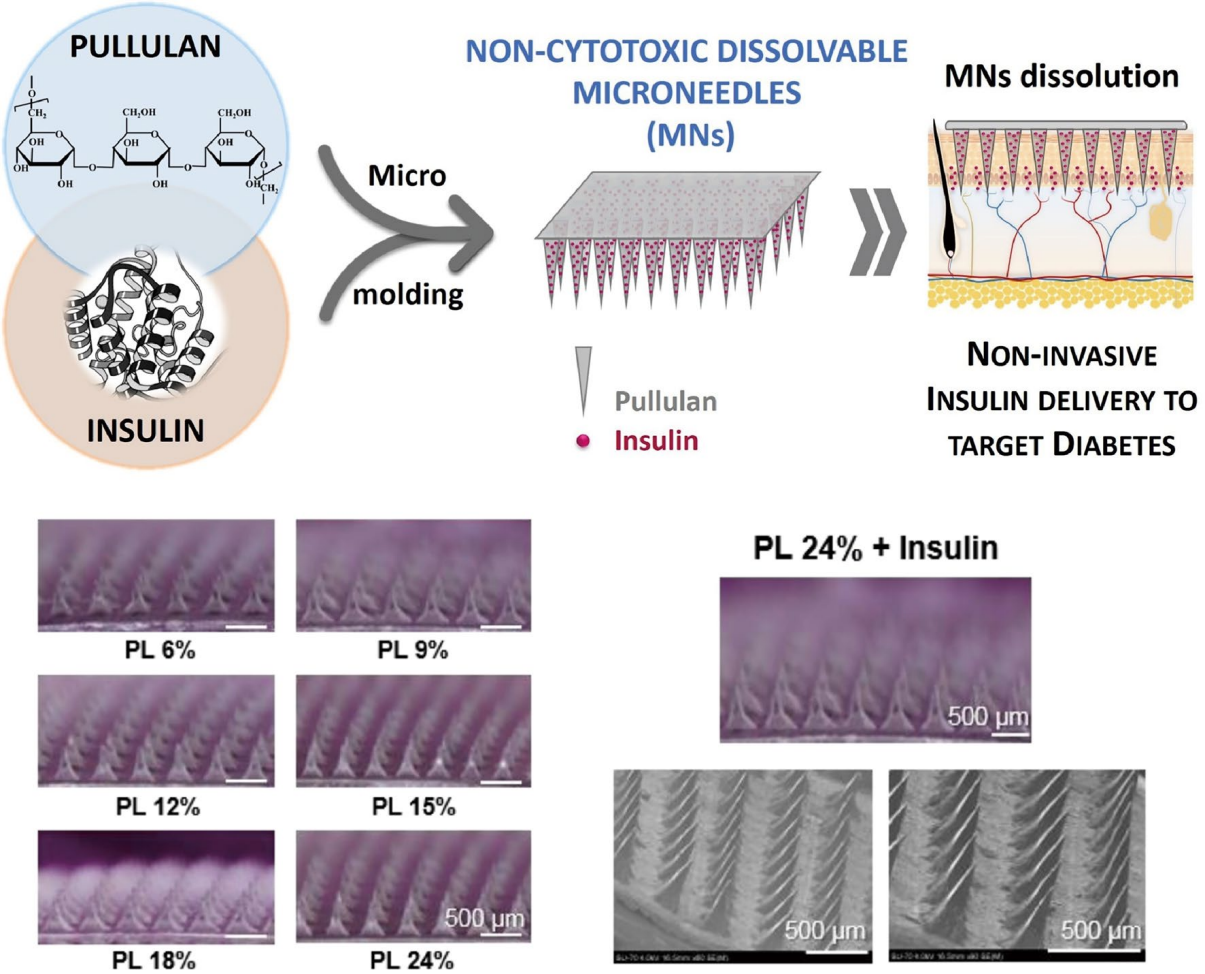
Pullulan. Reproduced with Permission [110]. Copyright 2020, Elsevier Ltd. Patch-form microneedles derived from pullulan, designed to enhance the transdermal absorption of insulin. Reproduced with Permission [110]. Copyright 2020, Elsevier Ltd

Pullulan, another hydrophilic linear polymer typically obtained from the fungus *Aureobasidium pullulans*, is composed of maltotriose units linked by α-1,6 glycosidic bonds [105]. Its surface contains abundant hydroxyl groups, which facilitate chemical modification [106]. Pullulan and its derivatives, known for their excellent film-forming abilities, adhesiveness, and mechanical properties, have been used to fabricate MNs for enhanced transdermal delivery of pharmaceutical molecules [107–110]. Fonseca et al. [110] used micromolding to produce pullulan-based MNs for insulin delivery (Fig. 8). These MNs dissolved in the skin within 120 min post-insertion, delivering 87% of the insulin dose. Vora et al. [108] developed dissolvable pullulan MNs for the transdermal delivery of small molecules (methylene blue and FS) and biomolecules such as FITC-labeled bovine serum albumin and insulin. Ex vivo permeation studies using porcine skin demonstrated 95%–100% dissolution of these MNs, similar to the efficacy observed with solid MNs, which suggests their potential for clinical applications.

### Cellulose

**Fig. 9:**
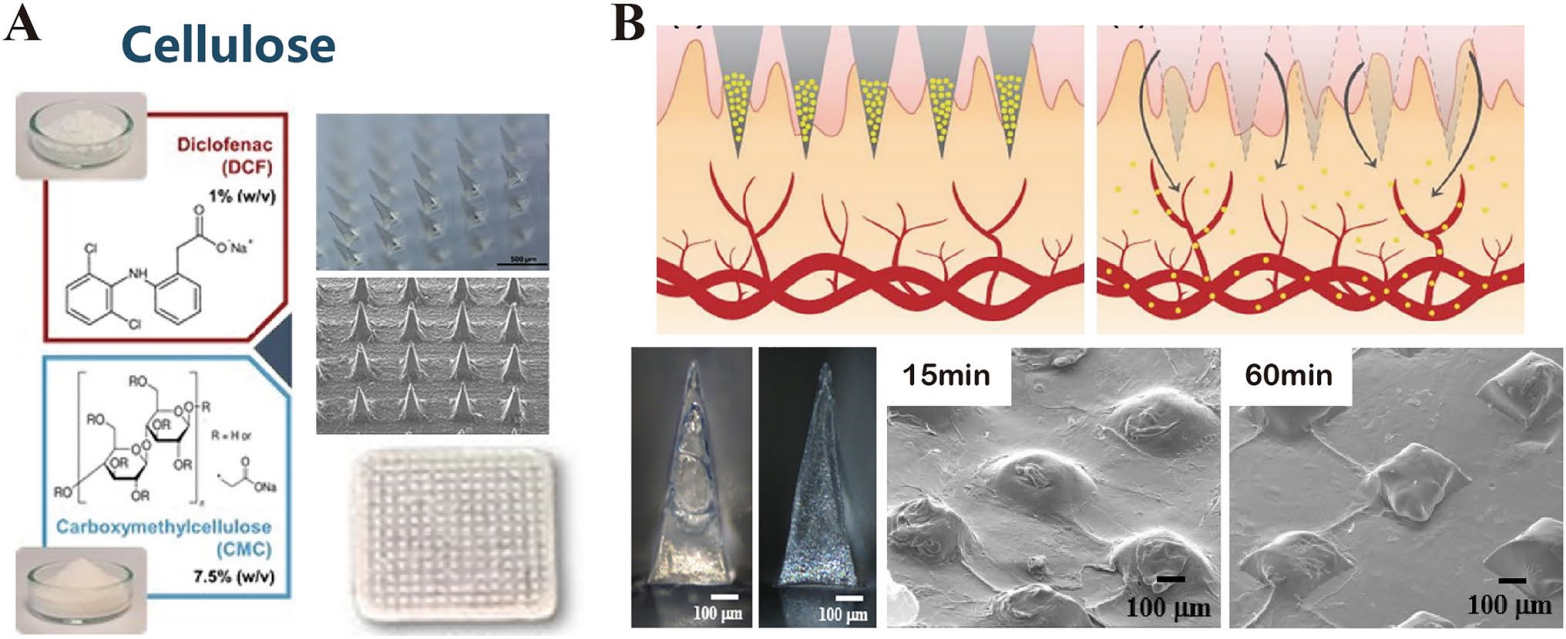
**A** Cellulose. Reproduced under the terms of the Creative Commons Attribution-NonCommercial-NoDerivs License [112]. Copyright 2022, The Authors, published by Wiley–VCH GmbH. **B** Carboxymethyl cellulose-based, tip-loaded dissolving microneedles, developed for the transdermal administration of donepezil hydrochloride, aiming to improve Alzheimer’s disease therapy. Reproduced with Permission [115]. Copyright 2016, Elsevier B.V

Cellulose, a polysaccharide with its glucose monomers interconnected by β-(1,4)-glycosidic bonds, is abundant in wood, cotton, bacteria, and algae (Fig. 9A) [111, 112]. Cellulose-based MNs have been used in cancer therapeutics [107, 113]. Cellulose derivatives such as CMC and hydroxypropyl methylcellulose (HPMC) are insoluble in water and conventional organic solvents at ambient temperatures [114]. Hence, these derivatives are widely recognized in biomedicine for their hydrophilicity and are employed as thickeners, adhesives, stabilizers, and film formers. Kim et al. [115] developed HPMC-based MNs encapsulating donepezil hydrochloride (DPH), an acetylcholinesterase inhibitor, for Alzheimer’s disease management. Remarkably, they achieved a high DPH encapsulation efficiency of 78% w/w and demonstrated rapid release, with over 95% of the drug delivered within 5 min after insertion. These MNs also exhibited adequate mechanical strength for skin penetration and dissolved entirely within 15 min (Fig. 9B) [115]. Lan et al. [116] used CMC to deliver a range of chemotherapeutic and immunostimulatory agents, including anthracycline drugs such as DOX and idarubicin, for skin cancer therapy. However, CMC-based MNs displayed a relatively slower dissolution profile than other polysaccharides, such as HA, which typically dissolve completely within approximately 60 min. Nevertheless, this dissolution rate can be significantly accelerated by combining CMC with other substances such as arginine heptagluconate and sucrose.

Innovative approaches have been proposed to optimize the properties of CMC in MN formulations. Lee et al. [117] created MNs with a CMC-trehalose matrix, in which the trehalose component expedited dissolution without compromising the structural integrity provided by CMC. Similarly, Park et al. [118] enhanced both the mechanical strength and dissolution kinetics of MNs by combining CMC with pullulan, thereby facilitating controlled drug release. To address the inherent brittleness of CMC, Qiang et al. [119] introduced polyvinylpyrrolidone (PVP) into a CMC matrix to produce a composite material with superior mechanical behavior and thermal stability. The 10% PVP/2% CMC-Na composite was the optimal formulation that displayed optimal surface morphology, higher degradation, and a higher melting point, indicating its potential for advanced MN applications.

### Chondroitin Sulfate

**Fig. 10:**
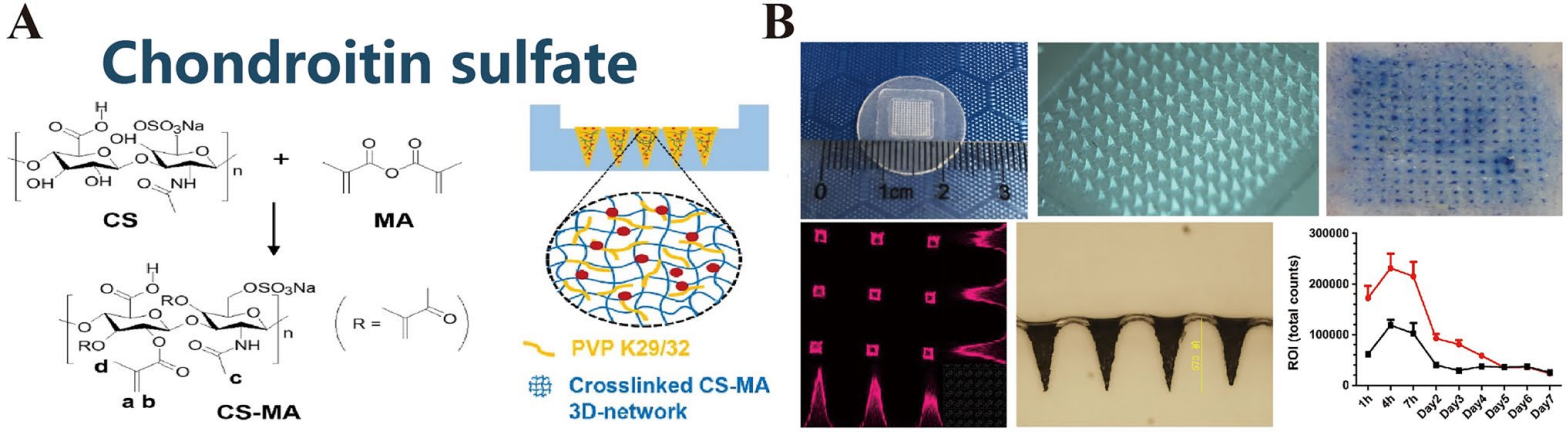
**A** Chondroitin sulfate. Reproduced with Permission [121]. Copyright 2022, Elsevier B.V. **B** Microneedle formulation containing chondroitin sulfate that dissolves to offer significant safeguard against life-threatening enterotoxin exposure. Reproduced under the CC BY-NC-ND license [123]. Copyright 2019, The Authors, published by Elsevier Ltd

Chondroitin sulfate is a sulfated GAG with repeated disaccharide units, each consisting of *N*-acetylgalactosamine and d-glucuronic acid linked by β-(1,3)-glycosidic bonds (Fig. 10A) [120, 121]. It is a major component of animal connective tissue and extracellular matrix, and its structure and properties vary significantly with the pattern of sulfate substitution along the carbohydrate chain [122]. Moreover, its multiple surface hydroxyl groups make it highly water-soluble and an excellent candidate for the preparation of DMNs.

Fukushima et al. [124] reported that chondroitin-sulfate-enriched MNs exhibited superior bioavailability compared to dextran MNs when both transdermal systems were used to administer recombinant human growth hormone (rhGH) and desmopressin (DDAVP) in a rat model. Chondroitin sulfate-based MNs afforded an impressive 95% bioavailability of the drugs, exceeding the 73% achieved using dextran MNs. Additionally, these MNs maintained the stability of rhGH and DDAVP over a month at storage conditions of − 80 and 4 °C. Poirier et al. [125] fabricated MNs using a blend of chondroitin sulfate and hydroxyethyl starch. These MNs largely preserved the antigenicity of the hepatitis B surface antigen (HBsAg) for 6 months at 37 and 45 °C, with a minimal degradation of 10% observed at 50 °C. Another innovative application of chondroitin sulfate-based MNs was immunization with recombinant Staphylococcus enterotoxin B protein (rSEB), which markedly extended the antigen retention time in vivo (Fig. 10B) [123]. These studies highlight the potential applications of chondroitin sulfate-based MNs as highly attractive substitutes for conventional hypodermic injections, particularly vaccine delivery.

### Bletilla Striata Polysaccharide

**Fig. 11:**
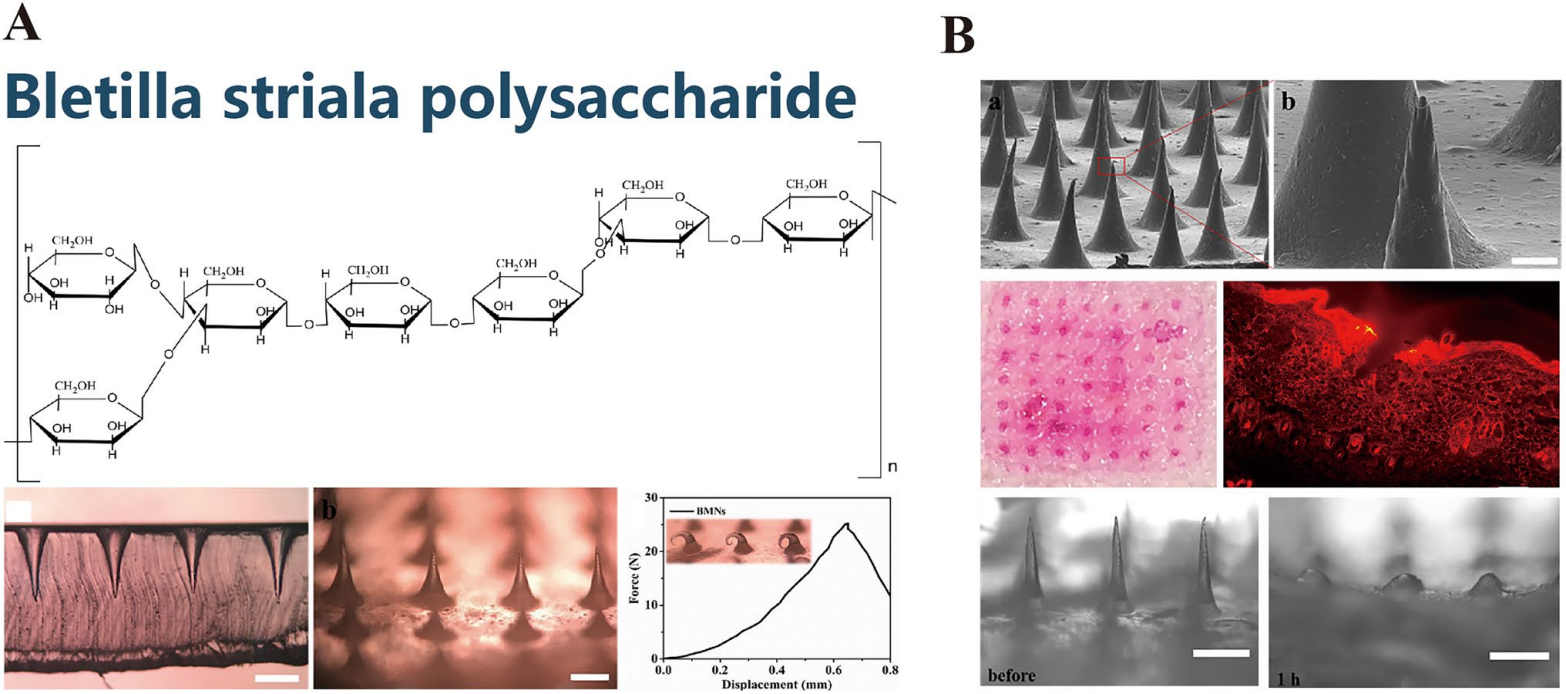
**A** *Bletilla striata* polysaccharide. Reproduced with Permission [129]. Copyright 2018, Elsevier B.V. **B** *Bletilla striata* polysaccharide-based microneedles designed for the effective delivery of prototype pharmaceutical compounds. Reproduced with Permission [129]. Copyright 2018, Elsevier B.V

*Bletilla striata* polysaccharide (BSP), a water-soluble polysaccharide extracted from the orchid *B. striata*, comprises monosaccharide units of α-mannose, β-mannose, and β-glucose [126, 127]. It is traditionally employed in medicine for its hemostatic, wound-healing, antimicrobial, and anti-inflammatory properties [128]. However, it has recently been used in the development of MNs for the delivery of bioactive molecules. Hu et al. [129] pioneered the use of BSP MNs as potential drug delivery systems (Fig. 11A and B). They used BSP-based dissolvable MNs for the delivery of rhodamine B (RhB), a model drug, and revealed their favorable moldability and biocompatibility. These MNs completely dissolved in murine skin within 1 h post-insertion. In vitro permeation experiments indicated that BSP MNs released 731.19 ± 75.52 and 389.95 ± 51.48 μg of RhB at 10 and 5 wt%, respectively, over 4 days.

The transition from BSP to another notable herbal polysaccharide, panax notoginseng polysaccharide (PNP), has emerged from the rich pharmacopeia of traditional Chinese medicine (TCM) [130]. PNP showed enhanced carrier potential owing to its ability to interact with both hydrophilic and lipophilic molecules [131]. Wang et al. [131] revealed that PNP can significantly induce maturation in bone marrow dendritic cells and elicit T-cell mediated immune responses through the TLR4-mediated NF-κB signaling pathway, which highlights its potential as an adjuvant in transdermal immunization. Moreover, these PNP-based MNs exhibited excellent mechanical strength and effective skin penetration, indicating their suitability for biomedical applications.

### Other Polysaccharides

Some polysaccharides, which have higher processing temperatures, pose challenges in MN fabrication, potentially leading to drug degradation or needle fracture [132]. Consequently, these polysaccharides are seldom used alone as MNs. However, the same polysaccharides may have potential auxiliary applications in MN-mediated drug delivery. For instance, sucrose is used as a protein stabilizer during MN preparation because of its thermal stability, which ensures the safe delivery of vaccines, such as influenza and malaria vaccines [133–135]. Zhu et al. [136] incorporated sucrose and trehalose in MNs and reported the maintenance of approximately 90% of the initial antigenicity of HBsAg, which was attributed to the protective function of these non-reducing disaccharides. Sucrose can also be used to enhance the stability of certain polymeric materials. Lee et al. [137] developed a porous MN patch with a backing layer. Sucrose was added to increase the stability of the PVP backing layer to prevent shrinkage or fracture during lyophilization.

Xanthan gum (XG), a high molecular weight polysaccharide gel, comprises a β-d-(1,4)-glucan backbone with side chains of β-d-glucuronic acid and β-d-(1,2)-mannose [138]. It is a nonionic polysaccharide and has poor solubility, low bioavailability, and difficulty in gel formation [139]. However, its carboxymethyl derivative (CMX) displays improved solubility and bioavailability and can form water-insoluble hydrogels upon crosslinking with metal cations owing to its anionic nature [140]. Although XG and its derivatives are rarely used alone to fabricate MNs, their excellent dispersion, moisturization, and solid particle suspension capabilities make them suitable thickeners for MN coatings [141–143].

Silk fibroin, derived from natural silk cocoons, is a novel slow-release material used for fabricating DMNs [144, 145]. Lee et al. [146] introduced highly flexible and porous silk fibroin-wrapped MNs to prevent neointimal hyperplasia. They assessed the cytotoxicity, controlled drug release, molecular permeability, vascular permeability, and tensile properties of the flexible MNs. The silk fibroin MNs conformed well to the delicate vascular tissue and exhibited good biocompatibility. Over a 28 days period, these MNs significantly inhibited neointimal hyperplasia, with a 62.1% reduction in new intimal formation. Silk fibroin MNs have also been used for the delivery of model drugs, including RhB, indocyanine green, and DOX, as well as insulin [147, 148]. Insulin was dissolved in Tris–HCl buffer (pH 7), and MNs were fabricated using photolithography by loading varying mass ratios of insulin, which validated the stability of insulin within the MNs. In addition, silk fibroin can be structurally modified. Yin et al. [149] modified silk fibroin with 2-ethoxyethanol to create novel expandable MNs. This modified silk fibroin exhibited controllable drug release ability compared to unmodified silk fibroin, and the improved swelling of the MNs was correlated with enhanced transdermal drug release kinetics and higher cumulative release rates.

In summary, polysaccharides extracted from natural sources have been extensively used to fabricate MNs, which have demonstrated excellent performance in various applications, including the treatment of ophthalmic diseases, drug delivery, vaccination, biosensing, gene therapy, and cancer treatment [150–153].

## Configurations and Drug Delivery Mechanisms of PSMNs

**Fig. 12:**
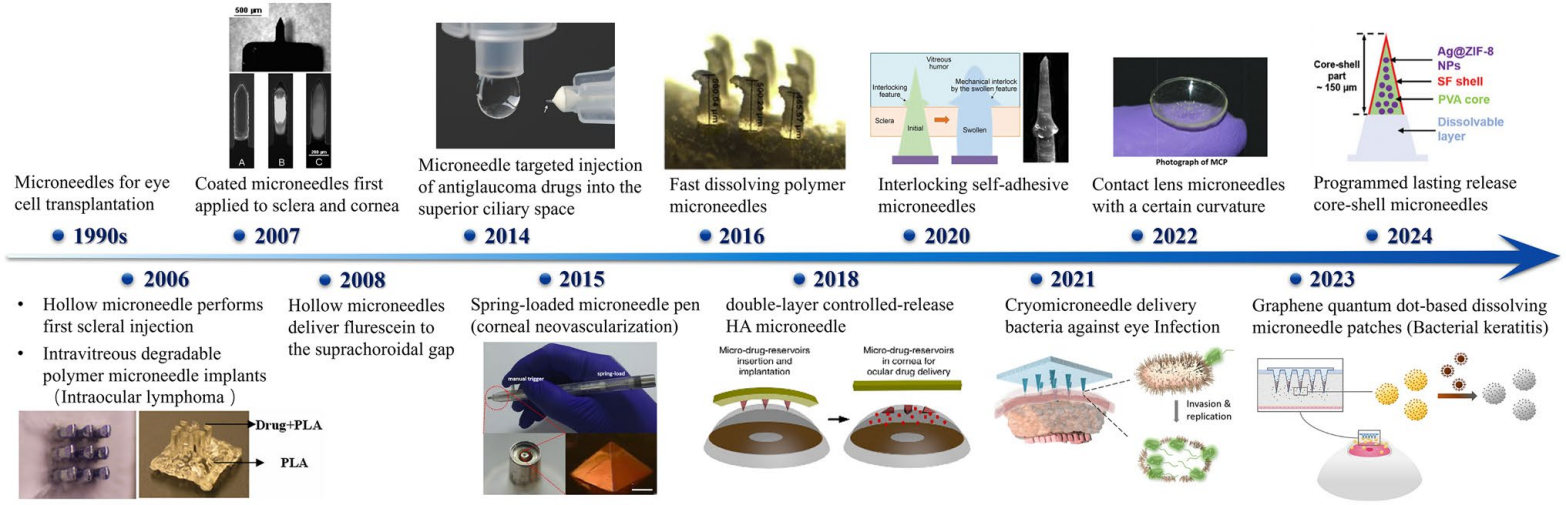
Evolution of microneedle applications in ophthalmic diagnosis and therapy. Reproduced with Permission [154]. Copyright 2006, Europe PMC. Reproduced with Permission [155]. Copyright 2007, Association for Research in Vision and Ophthalmology. Reproduced with Permission [156]. Copyright 2014, The Association for Research in Vision and Ophthalmology, Inc. Reproduced with Permission [157]. Copyright 2015, Elsevier B.V. Reproduced under the terms of the Creative Commons CC BY license [158]. Copyright 2016, The Author(s), published by Springer Nature. Reproduced under the terms of the Creative Commons CC BY license [50]. Copyright 2018, The Author(s), published by Springer Nature. Reproduced with Permission [159]. Copyright 2020 WILEY–VCH Verlag GmbH & Co. KGaA, Weinheim. Reproduced under the terms of the Creative Commons Attribution License [160]. Copyright 2021, The Authors, published by Wiley–VCH GmbH. Reproduced with Permission [161]. Copyright 2022 Elsevier B.V. Reproduced with Permission [162]. Copyright 2023 Elsevier B.V. Reproduced with Permission [163]. Copyright 2024 Wiley–VCH GmbH

Since its advent in the 1990s, MN technology has undergone substantial advancements, particularly revolutionizing ophthalmic surgery (Fig. 12). These advancements have been bolstered by concomitant progress in the fields of biology, materials science, and organic chemistry, which have collectively overcome the initial limitations of the MN size and shape. Consequently, an array of MN systems characterized by unique configurations and drug delivery mechanisms has been developed (Fig. 12). This review aims to catalog these MN configurations and systematically organize them based on their drug delivery mechanisms and structural attributes (DMNs, swelling microneedles (CMNs), solid-dose MNs (SDMNs), hollow MNs, and integrated MNs) to provide a comprehensive understanding of their evolution and current applications (Table 2).

**Table 2:** Comparative table of drug mechanisms

Mechanism type	Description	Advantages	Challenges
Dissolving	Materials dissolve rapidly in the skin to release drugs	Good biocompatibility, rapid drug release	Sensitive to environmental humidity, potential mechanical performance issues
Coated	Drug solution or dispersion coating applied on solid microneedles	Improved uniformity of the coating, maintains drug activity	Limited drug-loading capacity, potential biocompatibility issues with certain materials
Swelling	Hydrogels encapsulate drug molecules and facilitate controlled release through swelling behavior	Reduced infection risk, higher drug molecule loading capacity	Drug release affected by multiple factors, e.g., polymer molecular weight, swelling index
Solid dose	Direct delivery of drug molecules into the skin or tissues through physical penetration	Enhances bioavailability and distribution of drugs across the skin	Potential microbial infection risks, relatively low delivery efficiencies
Integrated	Biomimetic systems with diverse functionalities for drug delivery and biomonitoring	Multifunctionality, meets various requirements, synergistic outcomes	Technological complexity, need for precise control of manufacturing processes

### Dissolving Microneedles

**Fig. 13:**
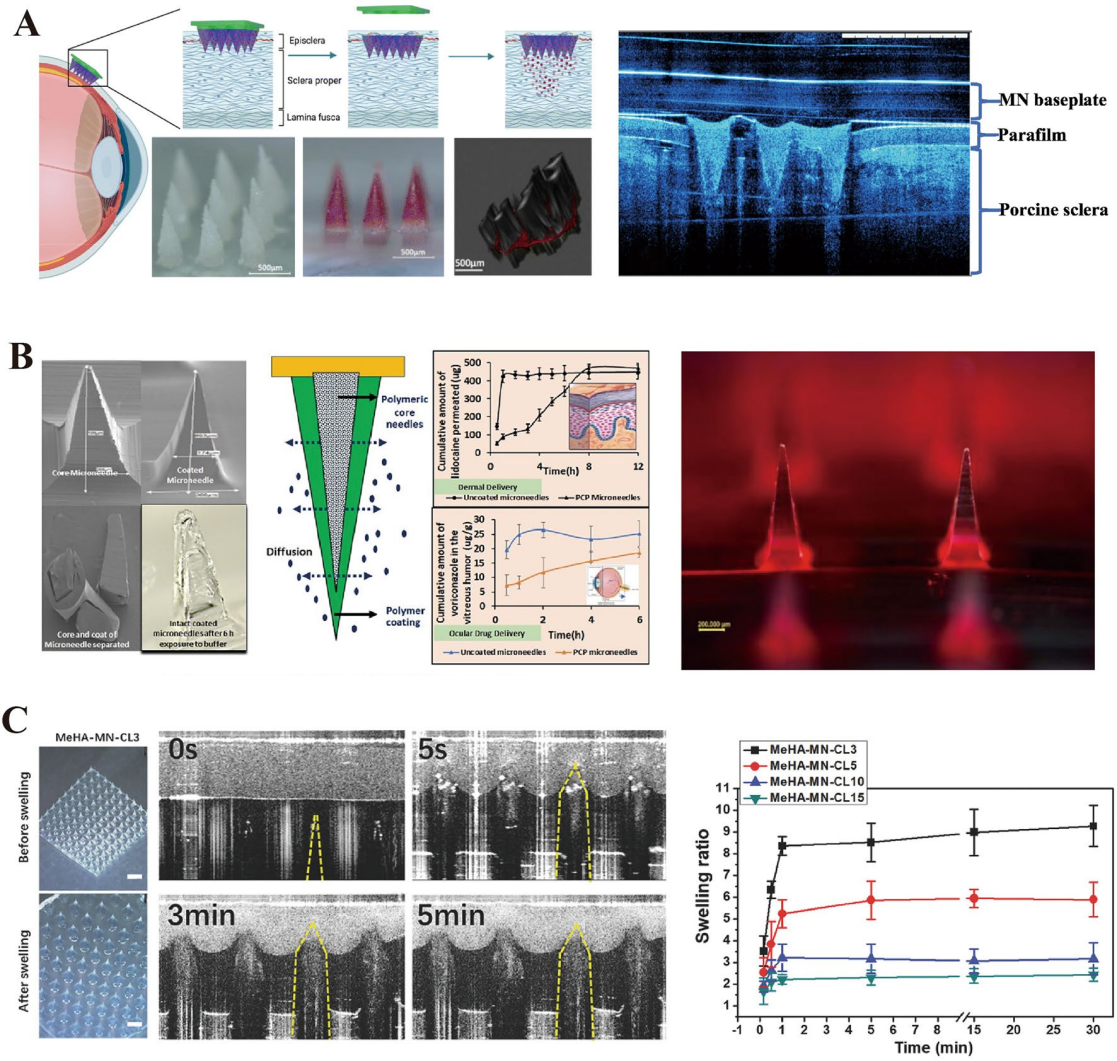
**A** Double-layer dissolving microneedles loaded with nanosuspension. Reproduced with Permission [165]. Copyright 2022, Elsevier B.V. **B** Coated polymer microneedles for controlled drug release. Reproduced with Permission [177]. Copyright 2022, American Pharmacists Association, published by Elsevier Inc. **C** Swelling microneedles that can extract ISF. Reproduced with Permission [178]. Copyright 2017, WILEY–VCH Verlag GmbH & Co. KGaA, Weinheim

DMNs are composed of soluble materials that dissolve rapidly in the skin and release drugs. These MNs typically contain natural polysaccharides, such as HA, trehalose, CS, and CMC, which are known for their excellent biocompatibility and biodegradability. The application of DMNs was first reported by Miyano et al. revealing their potential in the realm of drug delivery [164]. Wu et al. [165] developed DMN-containing nanosuspensions and successfully delivered hydrophobic drugs to the posterior segment of the eye (Fig. 13A). Furthermore, the dissolution rate of DMNs is a critical factor that influences drug release kinetics. By modulating the composition, concentration, or manufacturing process of the material, effective control of drug release dynamics can be achieved. McGrath et al. [166] created trehalose-based DMNs and found that their composition significantly affected their ability to penetrate the skin, with DMNs made from sodium alginate and CMC exhibiting high mechanical strength and effective tissue penetration. The self-dissolving nature, low toxicity, and cost efficiency of DMNs and the lack of tissue residue after application make them highly acceptable for clinical use. Moreover, the rapid dissolution of DMNs provides advantages in local anesthesia and vaccine delivery, enabling swift antigen release and simplifying administration protocols [167–169]. Moore et al. reviewed the application of DMNs in drug and vaccine delivery, emphasizing the importance of polysaccharides such as HA, trehalose, CS, and CMC in the fabrication of DMNs [170–174]. However, the sensitivity of DMNs to environmental moisture, which potentially leads to reduced mechanical performance, necessitates storage under dry and cool conditions, and the addition of stabilizers may be required to extend their stability and shelf life [175, 176].

### Coated Microneedles

Coated microneedles (CMNs) have been developed to address mechanical performance issues caused by the highly hygroscopic nature of DMNs [179, 180]. CMNs achieve drug release through the dissolution of a drug solution or dispersing coating applied on the surface of solid MNs, which can be made from materials such as silicon, metals, or polymers; the coatings are often composed of soluble polymers (e.g., PVA, PVP, and PLGA) and polysaccharides [155, 181]. Polysaccharide components, such as trehalose, dextran, CS, and sucrose, act as stabilizers, thickeners, and surfactants on the solid MN surface, improving the uniformity of the coating, maintaining drug activity, and ensuring stable storage and precise release of drugs. CMNs have been used for the delivery of DNA, proteins, peptides, and vaccines [182]. Chong et al. [183] successfully delivered therapeutic nucleic acid siRNAs to the skin using steel-coated MNs. Some examples of the diverse preparation methods for CMNs are inkjet (IJ) printing, wet etching, manual coating, dip coating, and spraying. Studies have indicated that reducing the MN surface area and increasing the coating viscosity can enhance drug encapsulation efficiency. Although CMNs have potential advantages in drug delivery, their limited drug-loading capacity restricts their use in certain applications. Furthermore, biocompatibility issues with silicon and other metals have prompted researchers to explore polymer-coated MNs. Shin et al. [184] prepared PLA-coated MNs with FITC-dextran and demonstrated their potential application in the protection and delivery of vaccines. Jakka et al. [177] compared the drug release rates of individual DMNs and PVP-coated MNs (Fig. 13B) and revealed that polymer-coated MNs delayed the release of active substances.

Overall, DMNs and CMNs, which are important MNs, exhibit tremendous potential and advantages for drug delivery. Future studies should aim to optimize the design and fabrication processes of MNs to enhance their drug-loading capacity, biocompatibility, and clinical efficacy.

### Swelling Microneedles

In biomaterial science, the modification and conversion of polysaccharide molecules are important strategies for developing state-of-the-art drug delivery systems. Through sophisticated chemical modifications, polysaccharides can be engineered into hydrogels with superior biocompatibility and biofunctionality [185]. These hydrogels, known as SMNs, not only mimic the hydration of biological tissues via intrinsic water absorption and swelling characteristics but can also be engineered for accelerated degradation under specific biocatalytic conditions, such as enzymatic reactions, thus allowing precise control of drug release kinetics [186–188].

SMNs, which have distinctive three-dimensional (3D) network structures, are a novel class of MNs for drug delivery. These networks can effectively encapsulate drug molecules and facilitate controlled release, which is mediated by the swelling behavior of the hydrogel or degradation of the polymer. This strategy was initially introduced by Donnelly et al. [189] in 2014 and has since been advanced in subsequent studies. The super-swelling polymer network developed by Donnelly et al. demonstrated an impressive drug delivery efficiency of up to 49% within 24 h, significantly broadening the applicability of transdermal delivery and revolutionizing conventional MN systems. Nonetheless, the drug release capabilities of SMNs are affected by several factors, including polymer molecular weight, swelling index, and crosslinker concentration. Modifying the crosslinker concentration can alter the crosslink density of the hydrogel, thus controlling its swelling behavior and drug release rate and enabling precise manipulation of drug release kinetics [190]. Moreover, the kinetics are influenced by the hydrogel pore size and the size of the drug molecules, paving the way for customized drug delivery solutions. Importantly, SMNs offer reduced infection risk and higher drug molecule-loading capacity than traditional DMNs, thus significantly enhancing drug delivery efficiency [191].

The diagnostic potential of SMNs is significant. Chang et al. [178] employed crosslinked MeHA MNs to extract interstitial fluid (ISF) from rodent models, achieving rapid and efficient glucose analysis within 10 min (Fig. 13C). This success not only optimized the sampling efficiency but also introduced novel pathways for swift diagnostics. Caffarel-Salvador et al. [192] expanded the use of SMNs in glucose sensing and insulin delivery by comparing the glucose concentration in ISF with that in plasma to validate the accuracy of SMN-collected glucose levels. This study confirmed the role of SMNs in minimally invasive patient monitoring and diagnostics.

In conclusion, SMNs are versatile tools for both drug delivery and diagnostics, and promise extensive future applications in biomedicine. Future studies should concentrate on refining hydrogel preparation techniques, improving drug-loading efficiencies, and broadening the scope of biomedical applications to offer safer, more effective, and more convenient therapeutic options for patients. Furthermore, an in-depth study of hydrogel biodegradation and drug release kinetics will advance personalized medicine by facilitating targeted and individualized drug delivery approaches, potentially revolutionizing clinical therapies.

### Solid Dose Microneedles

SDMNs are widely employed in passive diffusion and pressure-mediated drug delivery because of their unique design and functionality. SDMNs encompass solid and hollow MNs, each of which leverages different mechanisms for effective drug administration.

**Fig. 14:**
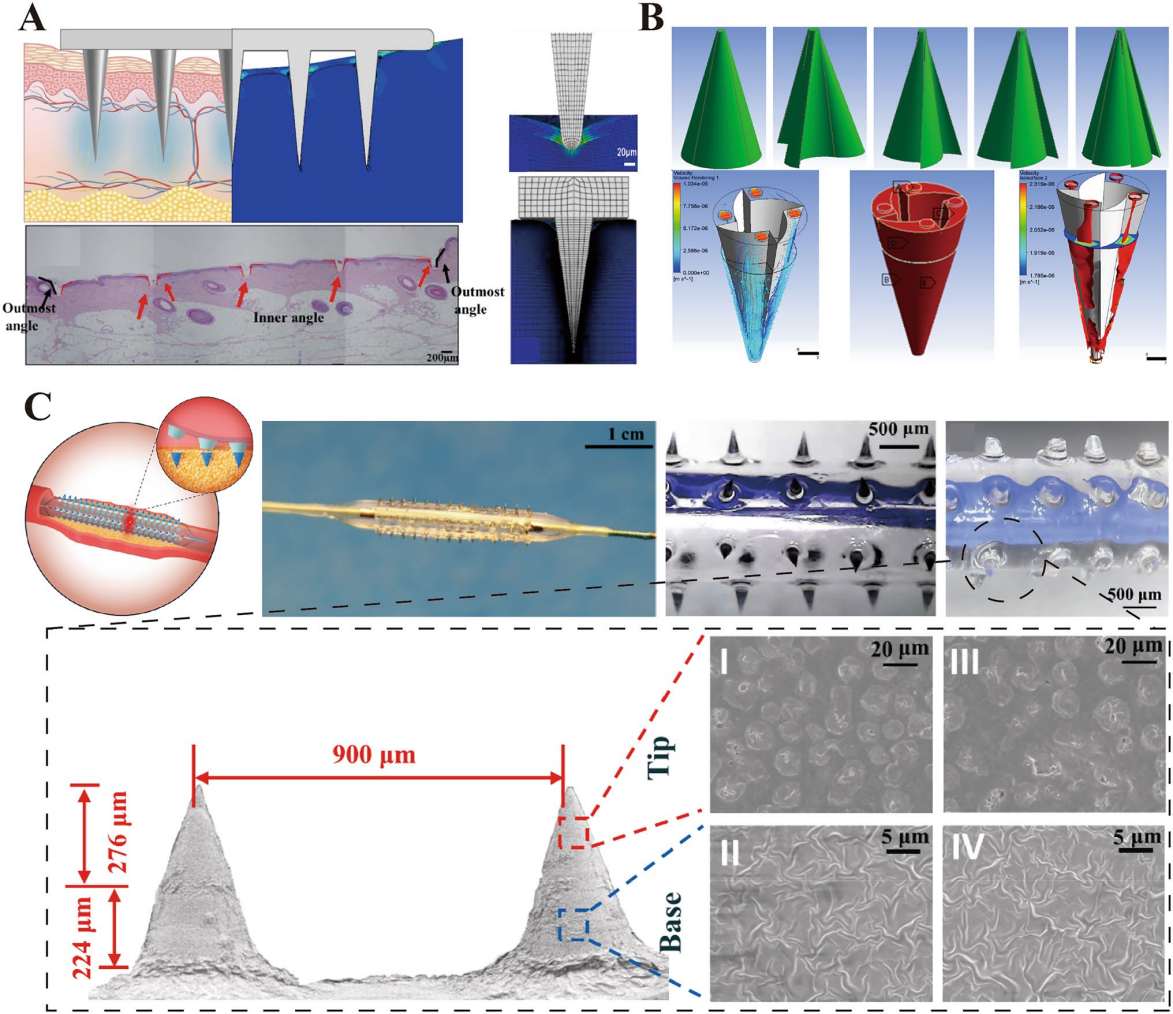
**A** Solid microneedles simulated using finite element method. Reproduced under the terms of the Creative Commons CC-BY license [200]. Copyright 2021, The Author(s), published by Elsevier Ltd on behalf of Acta Materialia Inc. **B** Serpentine-shaped semi-solid microneedle with grooves. Reproduced with Permission [201]. Copyright 2021, Elsevier Ltd. **C** Integrated microneedles integrated with drug elution balloons. Reproduced under the CC BY-NC-ND license [202]. Copyright 2022, The Authors, publishing services by Elsevier B.V. on behalf of KeAi Communications Co. Ltd

Solid MNs deliver drug molecules directly into the skin or other organ tissues through physical penetration without altering the needle form [193]. Upon breaching the outer layer of the skin, these needles create microchannels in the dermis, enhancing the bioavailability and distribution of drugs across the skin, thus optimizing drug administration efficacy. Studies have shown that solid MNs significantly enhance transdermal absorption in vitro, improving the bioavailability and kinetics of transdermal transport [194]. Compared with intramuscular injections, solid MNs exhibit a longer duration and a more intense antibody response during vaccine delivery [195]. Typically, these needles are fabricated from materials such as silicon, metals (such as titanium or stainless steel), and specific polymers [196–199]. Researchers have employed advanced methods, such as finite element analysis, to delve into the mechanisms of solid conical MN array skin penetration, revealing their mechanical stability and penetration capabilities (Fig. 14A) [200]. Nevertheless, solid MNs have limitations in clinical applications, such as potential microbial infection risks and relatively low delivery efficiencies.

Hollow MNs were developed to address these challenges. Each needle tip of a hollow MN is designed with a cavity to transport drug solutions to target sites through passive diffusion mechanisms such as pressure, electrotransport, and other stimulus-responsive methods [203]. Hollow MNs have extensive applications in drug delivery, insulin and vaccine delivery, biomonitoring, cancer therapy, and ocular drug administration. Patel et al. [204] successfully injected nanoparticle and microparticle suspensions into the choroidal space for reliable posterior eye segment drug delivery using hollow MNs. They used 800–1000-μm-long needles with an applied pressure of 250–300 kPa, which allowed optimal injection volumes of 15–35 μL into the suprachoroidal space. Furthermore, the design parameters of hollow MNs, such as internal diameter, tip size, applied pressure, insertion and retraction depths, and length, affect the drug flow rate and delivery efficiency. Similar to subcutaneous injection needles, hollow MNs can control the rate of drug release by adjusting process parameters, such as aspect ratio, which facilitate rapid release, slow infusion, and time-dependent administration efficiencies. Maaden et al. [205] developed an insertion device and an automated injection pump system capable of precisely controlling the injection process, including injection depth, volume, and infusion speed, thereby adjusting the drug delivery rate. However, hollow MNs have relatively weak mechanical strength, and careful attention is required in designing the needle for insertion [194]. Moreover, issues such as leakage and clogging during injection can limit the scope of their applications.

To overcome these limitations, Ahmad et al. [201] proposed a novel hollow polymer MN design that combines the features of biodegradable polymer MNs with those of hollow MNs (Fig. 14B). This design features semi-solid, fang-like conical grooves around the hollow needle, enhancing mechanical strength, enabling immediate insulin injection, and minimizing eddy current formation. The needle body is fabricated from biocompatible polymers and equipped with pH-responsive drug carriers to facilitate ISF extraction and long-term insulin release. This design effectively circumvents the drawbacks of traditional hollow MNs and shows great potential for insulin delivery and diagnostic applications.

In conclusion, SDMNs have significant advantages as efficient and safe drug delivery systems in both solid and hollow MN designs and applications. With continuous optimization of MN structural design, material selection, and precise control of the drug release rate and dosage, SDMNs are expected to play an increasingly important role in the future landscape of drug delivery.

### Integrated Microneedles

Researchers have developed biomimetic MN systems that emulate the structures and response mechanisms found in nature with diverse functionalities for drug delivery and biomonitoring [206]. Some examples are MNs developed for enhanced tissue adhesion, environmentally responsive MNs, and multifunctional MNs integrated with other devices.

Biomimetic MNs have been inspired by natural microstructures with high tissue adhesion, such as bee stingers, snake fangs, porcupine quills, eagle talons, and the teeth of eels [207–211]. Inspired by the flat and angled structures of shark teeth, Guo et al. [212] fabricated temperature-responsive hydrogel MNs capable of sensitive motion monitoring and controlled drug release by incorporating porous ordered structures. Their MNs-based device contained biomimetic structures with microfluidic and electronic devices and achieved diverse functionalities in wound management.

Similarly, to enhance the mucoadhesion of MNs in mucosal tissues, Zhu et al. [213] developed biomimetic suction cup-like multifunctional responsive drug-loaded MNs inspired by the predatory behavior of blue-ringed octopuses. Their Silk-Fp hydrogel MNs achieved efficient surface adhesion to mucosal tissues and highly efficient localized drug delivery. The inclusion of pNIPAM phase-change materials in the needle body allowed the MNs to contract in response to temperature after entering the target tissue, thereby actively injecting the drug solution. Furthermore, Pluronic F127 hydrogel-based suction cups were designed on Silk-Fp MNs, enabling microneedle patches (MNPs) to resist and remain stable in wet tissues for several days. Compared to other predominantly hydrophilic hydrogels, Silk-Fp MNs provide extended-release functionality and serve as more stable drug reservoirs to maintain a therapeutic effect.

Furthermore, the integration of MNs with drug-eluting balloons maximizes the advantages of PSMN devices for disease therapy. These integrated MNs have been used for treating vascular diseases such as atherosclerosis and arterial stenosis [214–217]. Huang et al. [202] developed a drug-loaded balloon with a series of photothermally triggered, sharp detachable tips for the treatment of atherosclerosis (Fig. 14C). Compared with traditional drug-eluting balloons, this integrated MN system has an extremely low drug loss rate, which significantly improves drug efficiency. This balloon drug delivery technology offers hope for the treatment of atherosclerosis and other diseases that require intraluminal targeted drug delivery.

In addition, 3D-printed MNs have been combined with other technologies, such as microfluidics, electrodes, and cell encapsulation, to develop novel MNs [218]. Stereolithography (SLA) and two-photon polymerization (TPP) are commonly combined with microfluidic technologies to fabricate injectable hollow MNs. Yeung et al. [219] used SLA 3D printing technology to create complex microfluidic MN devices that achieved the controllable injection of model drug solutions at varying flow rates. SLA-fabricated MNs can also be combined with carbon electrodes as biosensors for the in-situ monitoring of analytes. Miller et al. [220] used SLA to manufacture tetrahedral-shaped hollow MNs and manually inserted carbon electrodes into their internal structures to create real-time electrochemical sensing devices. These MNs exhibited high integrity and biocompatibility, and the integrated carbon electrodes could detect ascorbic acid and hydrogen peroxide. Zheng et al. [221] developed an integrated MeHA polymer MN array coupled with an electrochemical sensor for in situ, minimally invasive, and transdermal monitoring of various renal biomarkers in skin ISF.

In regenerative medicine, cell microencapsulation has been used to deliver cells directly to a target wound area, thereby promoting the healing process [222]. The integration of 3D-printed MNs with cell microcapsules paved a new direction in the development of MNs. Farias et al. [223] encapsulated human hepatocellular carcinoma (HepG2) cells in alginate capsules and used hollow MNs as delivery platforms. The experiments showed that the viability of HepG2 cells extruded from the device after 24 h was not significantly different from that of undisturbed control cells.

In summary, multifunctional integrated MNs represent a new trend in the development of PSMN technology [224]. Innovations in design and multifunctionality have met various requirements, leading to synergistic outcomes in biomedical applications. As technology continues to progress and innovate, the future application prospects of PSMN technology will broaden, offering more possibilities for disease treatment and health management [225, 226].

## Fabrication Techniques for PSMNs

**Fig. 15:**
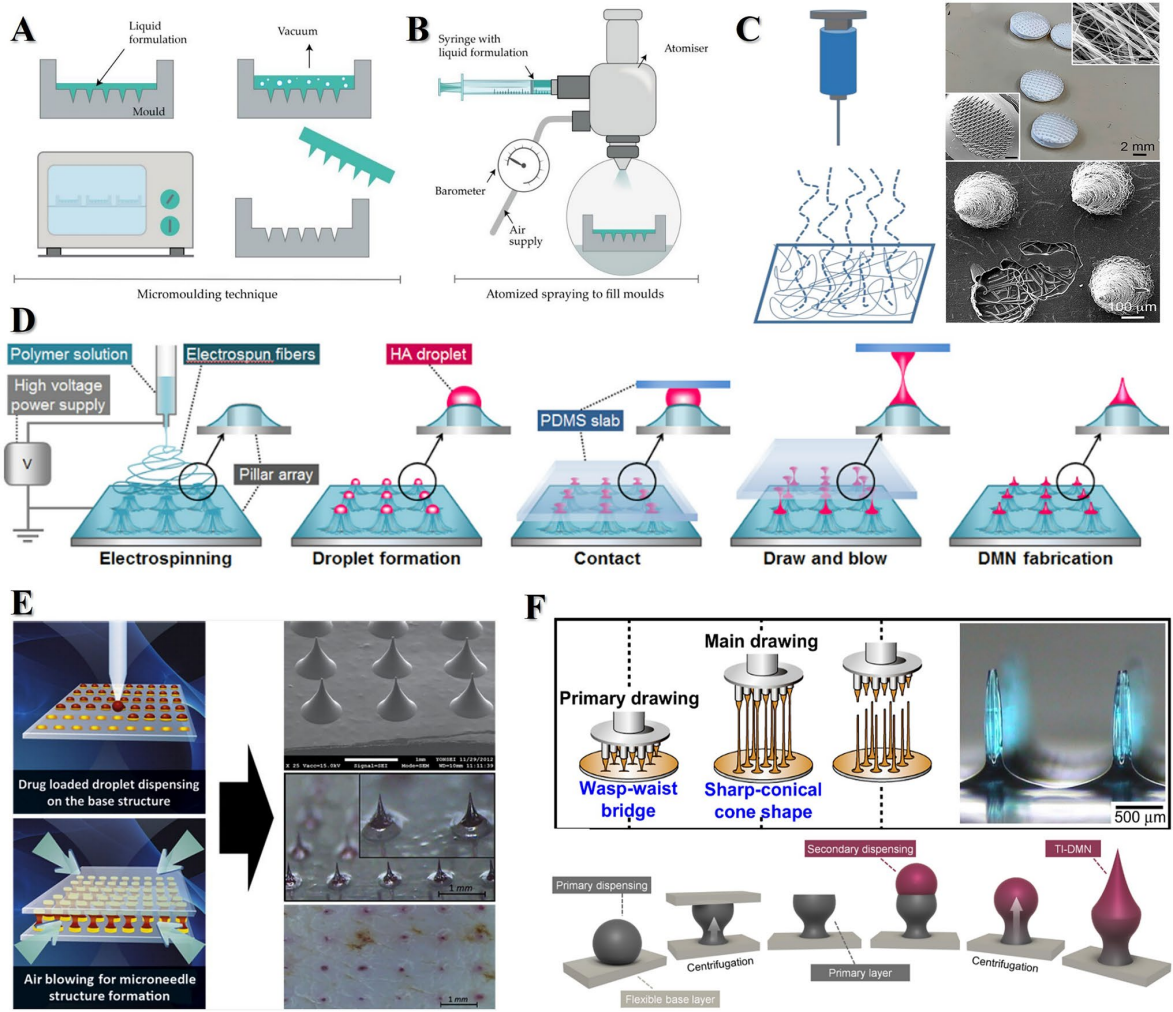
Fabrication techniques for PSMNs. **A** Micromolding. Reproduced under the terms and conditions of the Creative Commons Attribution (CC BY) license [227]. Copyright 2020, The Authors. Licensee MDPI, Basel, Switzerland. **B** Atomization spray coating. Reproduced under the terms and conditions of the Creative Commons Attribution (CC BY) license [227]. Copyright 2020, The Authors. Licensee MDPI, Basel, Switzerland. **C** Electrospinning. Reproduced with Permission [228]. Copyright 2020, American Chemical Society. **D** Electrospinning combined with drop-on-demand air blowing. Reproduced with Permission [229]. Copyright 2015, Elsevier Ltd. **E** Drop-on-demand air blowing. Reproduced with Permission [230]. Copyright 2013, Elsevier B.V. **F** Drawing lithography (top), Reproduced with Permission [231]. Copyright 2011, Elsevier Ltd. and Centrifugal lithography (bottom). Reproduced under the terms of the Creative Commons CC BY license [232]. Copyright 2019, The Author(s), published by Scientific Reports

The heterogeneity and tailorability of PSMNs technology make it promising for applications in ocular drug delivery systems. The crux of advancing this innovative technology is mastering its fabrication process. In this section, we summarize the predominant MN fabrication techniques, describing the unique attributes of various methodologies and their implications for the functional attributes of MNs (Fig. 15). We also systematically categorize MNs to elucidate recent advancements and potential applications in this burgeoning field. By delving into the nuanced preparation methodologies and taxonomy of MN types (micromolding, atomization spray coating, electrospinning, and electrospinning combined with drop-on-demand air blowing (DAB), DAB, drawing lithography, centrifugal lithography (CL), and 3D printing), this review article intends to provide invaluable insights and directives that will underpin future clinical investigations and pragmatic deployments of MN technology (Table 3).

**Table 3:** Comparative table of fabrication techniques

Fabrication technique	Description	Advantages	Challenges
Micromolding	Casting process using molds, typically made of PDMS	Simplicity, cost efficiency, high fidelity in MN production	Potential requirement for multiple vacuum cycles, material volume loss during mold curing
Atomization spray coating	Aerosolization of liquid polymer into a fine mist and drying	Scalability, suitable for various materials, including polysaccharides	Need to monitor viscosity and surface tension for optimization and reproducibility
Electrospinning	Elongation of polymer solution into nano- to microscale fibers using an electric field	Increased surface area, porosity, mimics extracellular matrix	Specific demands on solution properties, e.g., molecular weight, viscosity
Drop-on-demand air blowing	Precise deposition of polymer solution droplets followed by air blowing to form MNs	No need for heating or UV irradiation, suitable for high-viscosity solutions	Precise control over droplet deposition and airflow required for MN formation
Drawing lithography	Single-step molding alternative that avoids material loss during curing	Precision, high resolution, material compatibility, flexibility	Need for control over photopolymerization process for sharp and robust tips
Centrifugal lithography	Application of centrifugal force for precise control over microstructural shape and size	High uniformity of photoresist coating, suitable for complex substrates	Equipment and operational complexity compared to other methods
3D printing	Rapid and reproducible production of MNs with high precision and intricate designs	Customization of needle arrays, various printing methods available	High-resolution equipment can be expensive, material selection limited, potential cytotoxicity risks

### Micromolding

Micromolding is the principal fabrication strategy employed for generating PSMNs [233, 234]. This technique typically entails the use of polydimethylsiloxane (PDMS) owing to its exceptional thermal stability, pliability, and nonstick surface properties, which are conducive to the efficient release of MNs from the mold. Thus, PDMS has become the material of choice for mold creation in a multitude of device applications [235].

First, a master mold of the desired MN configuration is fabricated using robust materials, such as metal or silicon, via micromilling or photolithography. Then, this master mold is used as a template for casting the MNs [236, 237]. The casting process involves the infusion of a liquid prepolymer into mold cavities, which is facilitated by either vacuum application or centrifugation to ensure complete cavity filling. Subsequent solidification via drying or crosslinking reactions generates an MN array. The final demolding step releases the solidified MNs from the PDMS (Fig. 15A) [227].

The polymer curing phase is critical because it directly affects the mechanical strength of MNs. The choice of curing technique—thermal, drying, or ultraviolet (UV) light-induced—varies depending on the specific polymer material used [238, 239]. Although this micromolding method is known for its simplicity, cost efficiency, and high fidelity in producing functional and precise MNs, it is not devoid of challenges. One notable drawback is the potential requirement for multiple vacuum cycles to ensure the fidelity of the PSMN geometry.

### Atomization Spray Coating

Atomization spray coating is a novel fabrication technique for PSMN that employs aerosolization. This process involves the transformation of a liquid polymer into a fine mist via external forces such as ultrasonication, which is then directed into an MN mold through a nozzle connected to an air source. The subsequent drying phase solidified the polymer, forming the MN array (Fig. 15B) [227]. This method addresses manufacturing scalability challenges typically encountered in polysaccharide-based MNs, thereby enabling large-scale production. It is particularly adept at creating soluble MNs from diverse materials, including sugars, such as trehalose, dextran, and fructose, as well as polymers, such as PVA, PVP, CMC, HPMC, and sodium alginate [236].

The effectiveness of this technique was first demonstrated by McGrath et al. [166] who reported the successful fabrication of horizontal and laminated soluble MNs via atomization spray coating. Notably, they validated the suitability of this technique for generating MNs that can dissolve upon skin penetration and explored the influence of the atomization spray materials on the penetration depth of MNs. They observed that a layered spray approach, as opposed to a single spray application, resulted in MNs with denser structures composed solely of a single material. Therefore, this method not only improves the mechanical integrity of MNs but also their material compaction [240]. However, it is critical to monitor the viscosity and surface tension of the liquid polymer during fabrication to optimize PSMN formation and ensure reproducibility.

### Electrospinning

Electrospinning employs a syringe pump with a precision needle to direct a polymer solution toward a grounded collector [241]. This process is facilitated by an electrical field generated between the needle tip and collector, which elongates the solution into nano- to microscale fibrous scaffolds [242]. Electrospun nanofibers (ENFs) have garnered attention because of their increased surface area, porosity, and capacity to mimic the extracellular matrix, rendering them advantageous for wound dressing. These features have propelled ENFs to the forefront as a leading technology for creating cost-effective and versatile wound care products [243].

Polyvinyl alcohol (PVA), PVP, poly D, PLGA, polyurethane (PU), and polycaprolactone (PCL) are commonly used for electrospinning. Su et al. [228] developed a Janus antibacterial dressing from PCL and PVP, which combined an ENF membrane with a soluble MN array (Fig. 15C). This hybrid dressing leverages the porous structure of the fibers as a scaffold, enabling the electrostatic deposition of antibacterial peptides that target bacterial biofilms. The release kinetics of these peptides allowed sustained delivery for over 28 days. Yang et al. [229] extended the applications of electrospinning by incorporating polysaccharides. HA DMNs were produced on a PLGA micropillar array using droplet blowing (Fig. 15D) [229]. The inherent porosity of the array permitted rapid detachment and complete skin insertion of the DMNs, thus facilitating rapid implantation. In addition, the technology of fiber-reinforced silk fibroin microneedles has made significant strides by incorporating specific fibrous materials into the microneedles, thereby markedly enhancing their mechanical strength and tissue adhesion capabilities. For instance, Wang et al. [244] have engineered an innovative silk fibroin microneedle patch (SCEMN) that incorporates chitosan fibers, significantly enhancing the mechanical strength and tissue adhesion of the microneedles. This integration not only bolsters the stability and durability of the microneedles but also improves the efficiency and effectiveness of drug release through physical interlocking and bioadhesion mechanisms [245]. The incorporation of fibers introduces additional functionality into the three-dimensional structure of the microneedles, endowing them with superior performance in controlled drug delivery, as well as in antimicrobial and anti-inflammatory therapies. The fibrous reinforcement provides a multifunctional platform that can be tailored to specific therapeutic needs, offering a promising avenue for the advancement of transdermal drug delivery systems [246]. As this technology continues to evolve, it holds great promise for various biomedical applications, including but not limited to, enhanced wound healing, targeted drug delivery, and the treatment of localized infections [247, 248]. Future research and development efforts should focus on optimizing the fiber integration process and exploring the full spectrum of therapeutic applications for fiber-reinforced microneedles.

Electrospun ENFs made from polysaccharides such as HA, CS, starch, alginate, and chondroitin sulfate have also been used for wound dressing owing to their excellent wound-healing properties. Azar et al. [249] combined drug-loaded ENFs with MN arrays to achieve targeted drug delivery and extend drug release at the wound sites, thus promoting tissue regeneration and aiding the healing process of chronic wounds. This MN design also has the potential to enhance the mechanical stability and durability of needles.

Despite the versatility of electrospinning and its application in the creation of synthetic polymer-based MNs, the fabrication of PSMNs using this technique has been less explored. This limitation may be due to the specific demands of the electrospinning process, such as control over the molecular weight, viscosity, polymer concentration, and electrical conductivity of the solution, as well as the brittle nature of polysaccharides, which tend to absorb moisture, causing high surface tension during spraying and preventing rapid solvent evaporation. These factors present significant challenges in the production of precise and reproducible PSMNs devices from polysaccharide materials via electrospinning.

### Drop-On-Demand Air Blowing

The DAB technique represents an efficacious and gentle approach for the fabrication of PSMNs, obviating the requirement for heating or UV irradiation, which is prevalent in conventional methodologies [230]. This innovative technique enables the expeditious fabrication of DMNs in under 10 min at ambient temperatures ranging from 4 to 25 °C. The simplicity of the DAB process is underscored by minimal equipment demand.

The fabrication process entails the precise deposition of polymer solution droplets onto a flat substrate, followed by the application of a second substrate to the droplet array. The controlled upward motion of the latter substrate governed the height of the MN. Concurrently, the directed airflow thins and solidifies the droplets into the desired MN configuration. The subsequent separation of the two substrates yielded a bilaterally aligned array of MNs [250].

This method is particularly useful for the production of MNs from high-viscosity polysaccharide solutions. Kim et al. [230] demonstrated the versatility of the DAB technique by fabricating MNs with various concentrations of CMC (10%), HA (25%), and PVP (35%) (Fig. 15E). They comprehensively assessed the mechanical integrity, dissolution profile, and insulin release efficiency of the MNs. Their findings revealed that the DAB technique markedly preserved the bioactivity of insulin throughout the fabrication process, which highlights the potential widespread applications of this method for MN production. The MNs engineered using this method exhibited robust mechanical properties and efficient drug delivery to the skin, suggesting promising avenues for innovation in transdermal drug delivery systems.

### Drawing Lithography

The fabrication of PSMNs typically relies on multistep micromolding methods, which can lead to material volume loss during mold curing, necessitating repeated casting of the liquid polymer to maintain mold integrity. Drawing lithography is a rapid, single-step molding alternative that effectively circumvents the shortcomings of traditional micromolding.

Drawing lithography synergizes drawing and flatbed printing techniques, encompassing a variety of technologies such as electrospinning, magnetorheological lithography, thermal lithography, and CL [251]. Similar to the DAB method, this technology involves the photopolymerization of liquid droplet materials under short-wavelength light exposure, followed by a drawing process for chemical modification. Owing to its precision, high resolution, material compatibility, flexibility, and cost-efficiency, lithography has been widely adopted for the preparation of both solid and hollow PSMNs [252]. Li et al. proposed an innovative approach for fabricating 3D microstructures directly from 2D thermosetting polymers, bypassing the traditional molding steps [253]. They used stepwise controlled drawing to fabricate sharp DMNs directly from maltose (Fig. 15F) [231, 232]. This method achieves simultaneous molding and curing of MNs, overcoming the limitations of traditional microcasting methods.

Electrodrawing (ED) is another effective drawing lithography technique that employs the pyroelectric effect generated on polar dielectric crystals to induce an electric field around polymer droplets, invoking electrohydrodynamic (EHD) forces that deform the droplet into a Taylor cone shape. Vecchione et al. [254] successfully fabricated biodegradable polymer MNs suitable for subcutaneous injection using electrolithography. Additionally, researchers have proposed thermally driven drawing lithography techniques that incorporate pyroelectric substrates and magnetorheological drawing lithography (MRDL), which use magnetic fields to control the shape [255, 256]. Magnetic-drawing lithography is simple and, like thermal drawing, does not require masks, light exposure, or temperature control to fabricate complex and multiscale biomimetic MN structures.

CL combines the principles of traditional lithography with the application of centrifugal force, which is specifically tailored for microstructural fabrication, particularly at the nanoscale [257]. This technique enables precise control over the shape and size of the MNs while achieving high uniformity of the photoresist coating on complex or non-planar substrates. Huh et al. [258] compared the effects of two droplet-based DMN fabrication methods on the encapsulation and activity of an epidermal growth factor and ascorbic acid. Their findings indicated that the MNs produced via CL exhibited superior immunoreactivity and preserved drug activity, suggesting a potential advantage of the CL technique in manufacturing DMNs containing fragile biopharmaceuticals. Lee et al. [259] on confirmed the efficacy of CL in vaccinati. Using CMC as a structural polymer, they prepared lidocaine-containing DMNs using CL and verified their excellent local anesthetic effects. Lahiji et al. [232] employed CL to produce a novel tissue-interlocking DMN, incorporating a special design that improved skin penetration ability and drug delivery efficiency.

These studies demonstrate the tremendous potential of CL for fabricating efficient and precise drug delivery systems, marking significant advancements in the application of PSMN technology.

### 3D Printing

3D printing technology is a promising new method for fabricating PSMNs. This technique enables the rapid and reproducible production of MNs with high precision and intricate designs, while also allowing the customization of needle arrays based on specific shapes and dimensions, heralding a new era in MN fabrication. With continuous advancements in 3D printing technologies, an array of printing methods has emerged, including material extrusion-based fused deposition modeling (FDM), IJ printing, and light polymerization-based techniques such as SLA, digital light processing (DLP), TPP, and continuous liquid interface production (CLIP) [260].

**Fig. 16:**
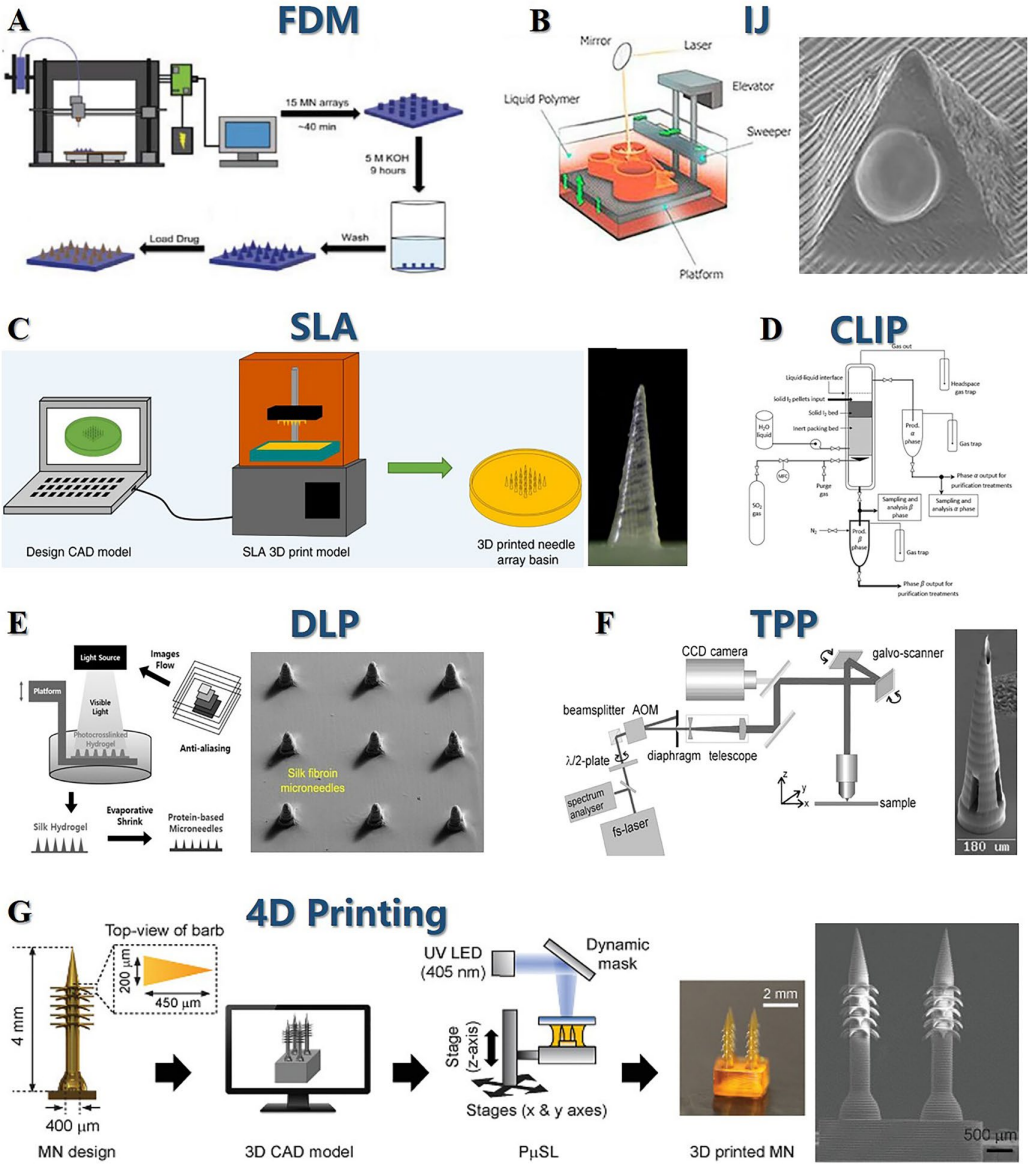
Process diagram, principle, and morphology of 3D printing for preparing microneedles. **A** Flow chart of fused deposition modeling (FDM) of microneedles. Reproduced under the terms of the Creative Commons Attribution License [263]. Copyright 2022, The Authors, published by Wiley–VCH GmbH. **B** Inkjet (IJ) printing equipment and needle tip morphology. Reproduced with Permission [265]. Copyright 2018, Elsevier B.V. **C** Stereolithography (SLA) printing equipment (left) (Reproduced under a Creative Commons Attribution 4.0 International License [266]. Copyright 2019, The Author(s), published by Springer Nature), and formed needle tip morphology (right) (Reproduced under the terms and conditions of the Creative Commons Attribution (CC BY) license [267]. Copyright 2020, The Authors. Licensee MDPI, Basel, Switzerland). **D** Principle of continuous liquid interface production (CLIP). Reproduced with Permission [268]. Copyright 2023, Elsevier B.V. **E** Digital light processing (DLP) printing principle and microneedle array. Reproduced with Permission [269]. Copyright 2020, The Korean Society of Industrial and Engineering Chemistry, published by Elsevier B.V. **F** Two-photon polymerization (TPP) printing principle. Reproduced with Permission [270]. Copyright 2007, Blackwell Publishing Ltd. **G** 4D printing process and microneedle array. Reproduced with Permission [271]. Copyright 2020, WILEY–VCH Verlag GmbH & Co. KGaA, Weinheim

FDM is a widely researched 3D printing method that constructs desired shapes by the layer-by-layer deposition of various thermoplastic polymers (such as PLA and thermoplastic PU) through a heated extrusion process [261, 262]. The material was heated to its melting point and extruded onto a building platform to form the required MN structure (Fig. 16A) [261, 263]. Shao et al. proposed an enhanced FDM strategy, loading a 3D printer with a biocompatible, water-soluble PU, aloe vera gel (avGel) and an MXene-doped conductive hydrogel to directly print biomimetic cactus-like MNs for tissue healing, controlled drug release, and wound motion monitoring [262]. These MNs not only demonstrated excellent rheological properties suitable for continuous printing but also exhibited good formability into complex shapes. Notably, by precisely controlling the extrusion speed of the hydrogel and movement of the printing head, they successfully printed a five-layer hydrogel scaffold with remarkable precision and structural integrity. Although FDM technology is relatively cost-effective, it is limited by print resolution and dimensional accuracy, often necessitating additional post-processing steps to smoothen the MN surface, thereby enhancing quality and performance [264].

IJ technology plays a pivotal role in the fabrication of PSMNs, particularly in the manufacturing of CMNs [265]. This technique involves the direct application of drug molecules to the surface of MN structures and has proven to be an effective method for the transdermal delivery of solid insulin [272]. In contrast to conventional nebulization spraying, IJ printing employs the piezoelectric effect to generate precise droplets through the formation of bubbles within the ink by heating the elements in the print head. Peer et al. [265] combined SLA and IJ printing to successfully fabricate insulin-CMNs (Fig. 16B) with mannitol, trehalose, and xylitol on their surfaces and achieved rapid in vitro release of insulin. Hilkiah-Ako et al. employed aerosol jet printing (AJP) to create DMNs composed of a PVP and trehalose blend [273]. They found that extremely high temperatures and slow flow rates can lead to nozzle clogging, where 40 °C was the optimal temperature for printing drug-loaded DMNs and 32 cm was the best flow rate. AJP offers greater flexibility with respect to aerosolization parameters, formulation ingredients, resolution, and handling of various formulation viscosities, making it suitable for the personalized production of MNs.

This technique has advanced rapidly owing to its low contamination rates, high reproducibility, cost-effectiveness, and minimal material wastage. Li et al. [274] merged drawing lithography with IJ printing to obtain a low-cost method for fabricating polymer MNs using direct-ink-writing 3D printing. They pneumatically extruded nanocomposite inks and stretched the droplets by adjusting the drawing parameters to generate sharp-tipped MNs on demand. This method is unconstrained by molds and, unlike drawing lithography, does not require external magnetic, mechanical, thermal, or electrical forces to maintain the structure during the solidification process, making it more straightforward and efficient. Moreover, the approach is compatible with various MNs based on soluble, biodegradable, and non-degradable polymers and can be used to fabricate multifunctional MN patches with heterogeneous integration, showcasing vast potential applications in MN technology.

Vat photopolymerization techniques are at the forefront of 3D printing technology because of their ability to produce PSMNs with high resolution. Advanced methods such as SLA, CLIP, DLP, and TPP that leverage the selective curing of liquid photosensitive resins with UV light or laser beams have been used for the precise fabrication of MNs.

For instance, SLA employs a UV laser to selectively cure liquid resins, thereby enabling the creation of objects with enhanced mechanical properties through selective photopolymerization. Krieger et al. devised an SLA-based approach for manufacturing MN molds with high-resolution tips (Fig. 16C) [266, 274]. SLA is instrumental in producing molds for MNs and is directly used for the large-scale production of solid MNs. Choo et al. [267] have refined key printing parameters, including the angle, height, aspect ratio, and spacing of the MNs (Fig. 16C) to yield high-resolution structures suitable for transdermal drug administration. CLIP, an iteration of SLA, allows the continuous and rapid production of high-precision MNs with smooth surface finishes, eliminating the need for layer-by-layer assembly (Fig. 16D) [268, 275, 276].

Light curing and printing technologies, particularly digital light processing (DLP), have become integral to the advancement of microneedle (MN) fabrication, offering a cutting-edge approach for creating polymeric solid microneedles (PSMNs). The precision and versatility of DLP make it an ideal candidate for the intricate and detailed construction of microneedles, which are essential for effective transdermal drug delivery systems. DLP technology operates by utilizing a digital micromirror device to selectively cure layers of photosensitive resin, one layer at a time, according to a predefined digital model. This process allows for the creation of microneedles with micron-level accuracy, enabling the design of complex geometries tailored to specific applications. The ability to produce multiple PSMNs simultaneously enhances efficiency and reduces production costs, making DLP an attractive option for scaling up microneedle production. One of the key advantages of using DLP for PSMNs is the material flexibility it offers. A wide range of photosensitive polymers can be employed, providing the opportunity to fine-tune the properties of the resulting microneedles, such as biocompatibility, mechanical strength, and degradation rate. This flexibility is crucial for adapting PSMNs to various medical and cosmetic applications, including drug delivery, cosmetic ingredient application, and diagnostic fluid sampling. Despite the numerous benefits, challenges remain in the development and application of DLP-prepared PSMNs. Material selection is critical, as it must balance biocompatibility with the necessary mechanical properties and curing behavior for effective microneedle performance. Scaling up the production process while maintaining the quality and consistency of the microneedles is another significant hurdle. Additionally, achieving regulatory approval for medical applications requires rigorous testing and compliance with industry standards. As research and development in the field of light curing and printing technologies continue to progress, it is expected that the sophistication and effectiveness of PSMNs will increase. The potential for these technologies to revolutionize transdermal delivery and diagnostics is immense, with ongoing advancements promising to unlock new possibilities for patient care and cosmetic treatments. DLP accelerates the process by simultaneously curing the entire layer with a projection light source. Shin et al. [269] pioneered the use of DLP to create protein-based MNs (Fig. 16E), specifically silk fibroin MNs, to assess their capacity for transdermal drug delivery.

TPP, which uses a femtosecond laser to stimulate a photoinitiator for a photochemical reaction, is ideal for crafting complex and detailed hollow MN structures (Fig. 16F) [270]. Despite the exceptional resolution of TPP, its slower production rates and requirement for expensive equipment are notable drawbacks. Additionally, the reliance on photoinitiators in photopolymerization methods poses potential cytotoxicity risks. In contrast, FDM provides a wider choice of materials, potentially circumventing the use of toxic substances.

As 3D printing technology evolves, 4D printing has also emerged in PSMN manufacturing, adding a time dimension that allows smart materials to react to environmental stimuli, such as light, magnetism, electricity, temperature, humidity, and pH, altering their shapes and functions. Han et al. [271] exploited deswelling deformation from graded crosslinking in light-cured polymers and 4D-printed biomimetic MNs with barbs (Fig. 16G). They meticulously controlled the barb thickness and curvature by adjusting light exposure and precursor solution composition, resulting in MNs with augmented tissue adhesion. This innovative approach opens new avenues for MN applications, highlighting the expansive potential of 4D printing in smart drug delivery systems.

The confluence of 3D printing technology with ancillary techniques such as electric field assistance, magnetic field manipulation, and temperature control has paved the way for the creation of PSMNs with integrated multifunctionality [277]. The development of MNs that integrate real-time biosensors has significantly expanded the scope of PSMNs applications. Despite the considerable potential of 3D printing for PSMN fabrication, the technology has both technical and material constraints [278]. High resolution is imperative to ensure the definition and effectiveness of MNs; however, 3D printers capable of such precision are often prohibitively expensive and operationally time-intensive. Currently, the reliance on photosensitive polymers to create sharp and mechanically robust tips limits the selection of materials, especially polysaccharides. Polysaccharides are typically processed into coated or dissolvable PSMNs via spraying or extrusion, which may lead to clogged nozzles or compromise drug integrity. To overcome these obstacles, 3D printing technology must be enhanced and novel materials and methodologies must be explored for more efficient, economical, and accurate PSMN production. For this, new biocompatible and biodegradable polysaccharide materials must be developed, printing parameters must be fine-tuned to reduce the risk of nozzle clogging, and innovative techniques must be incorporated to augment PSMN functionalities and performance. These 3D-printed PSMNs are expected to play an increasingly vital role in future medical and healthcare applications.

The rapid development of 3D printing technology in recent years has made significant progress in both technology and materials, offering hope for its widespread application in the manufacturing of microneedles in the future. In terms of technology, companies such as Stratasys and Shenzhen Mofang Precision, which are domestic and international 3D printer R&D companies, have continuously broken through the bottleneck of precision manufacturing. They have achieved high-precision stereolithography at the micro-nano scale, with optical precision up to 2 μm. This not only allows for the manufacturing of micrometer-scale microneedles but also makes the production of slanted, multi-layered, and porous special-shaped microneedles possible [279]. In terms of materials, 3D printing inks have evolved to include high-strength, high-hardness, high-temperature resistant, tough, and biocompatible resins. Biocompatible resins are already widely used in the manufacturing of food, dental, and medical devices, meeting the needs of microneedle manufacturing. Furthermore, by combining 3D printed microneedles with template molding techniques, the manufacturing of microneedles will no longer be limited to 3D printing materials, allowing for a broader range of biocompatible materials to be used in microneedle production.

### Characterization Methods for MNs

Characterization of MN is a multifaceted process that begins with the confirmation of their chemical structure, typically achieved through Fourier Transform infrared spectroscopy (FTIR) to identify specific chemical groups and bonds within the MN matrix. Thermogravimetric analysis (TGA) is then employed to assess the thermal stability and composition of the microneedles, providing insights into their material integrity. Morphological characterization, a pivotal aspect of MN characterization, is accomplished using scanning electron microscopy (SEM) and transmission electron microscopy (TEM), which offer detailed information on the surface topography and dimensions of the microneedles. Atomic force microscopy (AFM) further elucidates the height and surface roughness of the microneedles, contributing to a comprehensive understanding of their physical attributes. The size distribution and stability of microneedles are evaluated through dynamic light scattering (DLS) and zeta potential measurements, which shed light on their behavior in solution. Additionally, nitrogen adsorption–desorption isotherms provide crucial information on the specific surface area and pore structure of the microneedles, which are vital for drug loading and release characteristics. Elemental mapping techniques enable the nanoscale visualization of the distribution of various elements within the microneedles, which is essential for understanding their composition and structure. X-ray diffraction (XRD) is utilized to determine the crystalline structure and phase composition of the microneedles, offering valuable insights into the physical stability and drug release profile of the material. Rheological testing evaluates the mechanical properties and viscoelastic behavior of microneedles, which is critical for predicting their performance in practical applications. Furthermore, for stimuli-responsive microneedles, such as those sensitive to pH or temperature, corresponding stimulus condition tests are imperative to ensure they exhibit the desired release behavior under specific physiological conditions. In summary, these characterization methods provide a comprehensive set of physical, chemical, and biological information for the design, optimization, and quality control of microneedles, ensuring the performance, safety, and efficacy of these products.

## Mechanisms of Polysaccharide-Mediated Ocular Drug Delivery

Polysaccharide-mediated ocular drug delivery mechanisms exert their effects through a multitude of pathways, which include: (1) leveraging the viscosity and bioadhesive properties of polysaccharides to prolong the residence time of drugs in the ocular environment; (2) forming nanocarriers to enhance drug delivery efficiency; (3) creating gels for sustained drug release; (4) utilizing the mucoadhesive characteristics of polysaccharides to extend the local action time of drugs; (5) serving as protective agents to prevent drug degradation; (6) chemically modifying polysaccharides to acquire specific physicochemical properties that improve drug delivery; (7) exploiting the thermosensitivity and pH-responsiveness of polysaccharides to regulate drug release; (8) combining with cell-penetrating peptides (CPPs) to enhance the ability of drugs to penetrate cell membranes; and (9) integrating with other drug delivery systems such as liposomes and nanoemulsions to improve drug stability and delivery efficiency. The integrated application of these mechanisms provides an effective strategy for enhancing the bioavailability and therapeutic efficacy of ocular drugs. By harnessing the unique attributes of polysaccharides, researchers can develop sophisticated ocular drug delivery systems that address current challenges and pave the way for more effective treatments.

Polysaccharide molecules are highly compatible with ocular tissues; hence, PSMNs have potential applications in the management of eye diseases. Polysaccharides offer natural and distinctive advantages for delivering medications to specific ocular tissues. Among ophthalmic formulations, polysaccharides represent a compelling choice for excipients because of their biocompatibility and adjustable biodegradability, which facilitate efficient local drug release and absorption [280]. The FDA has approved various polysaccharides for ophthalmic use, including alginate, cellulose, and its derivatives HA and XG. These molecules not only provide notable drug delivery benefits but also show considerable promise for the development of PSMNs. As an innovative transdermal delivery system, PSMNs offer a direct method of administering drugs to ocular tissues through their microscale structures. This method can potentially enhance drug bioavailability and therapeutic outcomes while reducing the risk of systemic side effects. In this section, we review the mechanism of action and significance of polysaccharide molecules in ocular drug delivery.

Ocular tissue is anatomically and functionally diverse, with different regions manifesting distinct pathophysiological traits. This diversity leads to variability in disease susceptibility and necessitates tailored therapeutic approaches [281]. The anterior segment, encompassing the cornea and conjunctiva, is vulnerable to environmental insults, potentially incurring abrasions, mild burns, and tear film dysfunction. Such afflictions can escalate to severe conditions, including cataracts, dry eye syndrome, glaucoma, corneal neovascularization, and various inflammatory disorders. Moreover, the posterior segment, comprising the retina and macula, is predominantly affected by diseases such as AMD and retinitis pigmentosa, which are the leading causes of visual decline and blindness [282].

This intricate ocular anatomy creates a unique milieu that presents formidable challenges for effective drug delivery. Therapeutic agents must traverse various static (e.g., the blood-ocular barrier), dynamic (e.g., tear fluid turnover), and metabolic (e.g., enzymatic degradation) ocular barriers [283]. The lipophilic nature of the corneal epithelium and its tight junctions impede hydrophilic drug permeation, whereas the structured, charged stroma poses a barrier to hydrophobic drug penetration. These barriers reduce the efficacy of conventional eye drops in achieving corneal penetration, thus restricting drug delivery to the intraocular tissues [284].

Polysaccharides and their derivatives are highly promising ophthalmic drug formulations. As early as 1930, Mott et al. [285] demonstrated the positive effects of *C. albicans* polysaccharides on ocular hypersensitivity, using them for sensitization and functional recovery experiments. Rolando et al. recently explored the use of tamarind seed polysaccharides in dry eye treatment, substantiating the therapeutic relevance of polysaccharides in ocular conditions [286, 287]. To optimize drug delivery to the eye, formulations frequently incorporate viscosity-enhancing polysaccharides, such as hydroxyethyl cellulose, hydroxypropyl cellulose, and HA, or diffusion enhancers, such as cyclodextrins, to augment diffusivity and bioavailability. Wu et al. [288] assessed mucoadhesive polymers derived from BSPs for the delivery of ofloxacin and confirmed their enhanced therapeutic effect on bacterial keratitis. Additionally, Zambito et al. [280] examined the use of polysaccharides as formulation agents in topical ophthalmic preparations, highlighting their potential to refine drug delivery modalities. Moreover, mucosa-adhesive polysaccharide nanoparticles or liposomes such as CS, HA, and alginate salts extend drug action through electrostatic interactions with ocular mucins [289]. Yu et al. [290] summarized the challenges and strategies for developing polysaccharide-based nanomaterials for ocular drug delivery, emphasizing the importance of structural stability and biological activity in fabricating efficacious drug carriers. Their views paved the way for more effective and safer novel treatments for ocular diseases.

HA, a naturally occurring polysaccharide, is beneficial for ocular applications [291]. It not only improves corneal hydration but also attenuates leukocyte migration to inflamed areas and fosters corneal wound healing, particularly through its interactions with the CD44 receptor [292, 293]. Its exemplary biocompatibility, mucoadhesion, antioxidative capacity, and non-immunogenic nature make it an optimal choice for tear substitutes and engineering drug delivery films and devices, particularly for managing dry eye syndrome [294–296]. HA has become the most widely used polysaccharide in the fabrication of PSMNs for treating ocular diseases owing to its vital role in the structural and functional integrity of the eye, combined with its high biocompatibility and superior drug release capabilities [56, 297]. However, its high solubility and rapid dissolution preclude sufficient mechanical strength for intraocular insertion when used alone. Hence, scientists have mixed and copolymerized HA with other biocompatible macromolecular materials for MNs with enhanced mechanical properties. Suriyaamporn et al. [55] fabricated rapidly dissolving PSMNs by combining HA with varying ratios of GAN loaded with 1% FS for the effective treatment of posterior segment eye diseases. Shi et al. [56] developed HA-based DMNs mixed with PLA to treat fungal keratitis. Albadr et al. combined HA with PVA/PVP to deliver amphotericin B (AMP-B), which proved to be an effective treatment against ocular fungal infections. In addition to blending with biocompatible polymers, HA can also be chemically modified to achieve the required mechanical strength and controlled drug release. Than et al. [50] fabricated a dual-release PSMN patch by methacrylating HA to treat corneal neovascularization. This modified HA exhibited extended protein drug encapsulation and release durations and biphasic drug release owing to its rapid dissolution.

Cellulose and its derivatives are important polymers used in topical ophthalmic formulations [298]. The insolubility of pure cellulose in water has necessitated the development of various soluble cellulose derivatives, including methylcellulose (MC), CMC, ethylcellulose (EC), hydroxyethylcellulose (HEC), hydroxypropyl cellulose (HPC), HPMC, and cellulose esters, such as cellulose acetate (CA) and cellulose acetate phthalate (CAP) [299]. These derivatives demonstrate enhanced solubility, viscoelasticity, and thermal stability, thereby presenting significant potential as drug carriers [300]. HPMC is a common ocular lubricant.

CS has emerged as a promising mucoadhesive polymer for the sustained release of ocular therapeutics [301]. The positive charge at physiological pH prolongs drug residence time via electrostatic interactions with negatively charged ocular mucins [302]. Furthermore, its inherent antimicrobial properties disrupt the integrity of bacterial cell walls. CS is proteolytically degradable into non-toxic byproducts, and its modifiable hydroxyl and amino groups enable tailorable physicochemical characteristics [7]. This versatility enables its application in a range of delivery modalities, including drug-loaded nanoparticles, liposomes, films, gels, and coatings for MNs. Karava et al. [303] notably advanced this field by developing CS-based nanocarriers containing dexamethasone (DM), sodium phosphate, and chloramphenicol, which have significantly enhanced drug stability, solubility, and mucosal permeation. Additionally, the inherent antimicrobial and mucoadhesive properties of CS enable the ocular applications of CS-based MNs.

Polysaccharides such as cellulose derivatives, XG, pectin, cyclodextrins, and alginate are thermosensitive and pH-responsive and exhibit ionic strength-dependent gelation [7, 304]. These in-situ gelling systems are at the forefront of extending ocular drug residence times. In this context, Kouchak et al. [305] developed a pH-responsive HPMC system that effectively delivers dorzolamide hydrochloride (DRZ) to manage intraocular pressure in glaucoma; this system enables gelation-enhancing mucosal adhesion without compromising corneal permeability.

In addition to natural macromolecules such as polysaccharides, artificial macromolecules, including DNA nanorobots and artificial peroxidases, have garnered significant attention due to their promising applications in the biomedical field [306–308]. These molecules boast notable advantages, such as high efficiency, multifunctionality, targeting capability, biocompatibility, and controllability. For instance, DNA nanorobots can specifically bind and silence c-Met gene expression, offering a novel strategy for cancer therapy. Artificial peroxidases, on the other hand, efficiently catalyze the production of reactive oxygen species to enhance tumor immunotherapy. Moreover, these macromolecules can be controlled by external factors like pH, temperature, or ultrasound, thereby improving the precision and safety of treatments. However, compared to polysaccharides, artificial macromolecules also have some drawbacks. Their stability within the complex in vivo environment may be compromised, leading to susceptibility to enzymatic degradation or aggregation. Despite their targeting capabilities, the efficiency of intracellular delivery remains a challenge that could affect therapeutic outcomes. Some artificial macromolecules may elicit immune responses, which not only limit therapeutic efficacy but also pose the risk of side effects. Production costs are also a critical factor; the complexity and high costs of synthetic processes may hinder their widespread application. Beyond these advantages and challenges, other considerations include the long-term effects and safety of artificial macromolecules, which need to be verified through clinical trials. Additionally, due to potential variations in individual patient responses to artificial macromolecules, personalized treatment regimens are necessary. In summary, while artificial macromolecules hold tremendous potential in the biomedical field, their clinical application requires overcoming challenges related to stability, delivery efficiency, immunogenicity, and controlling manufacturing costs. Future research should focus on these aspects to advance the development and application of artificial macromolecule therapeutics.

## Advancements in the PSMNs for Ophthalmological Diagnostics and Treatment

In 2007, Jiang et al. [155] demonstrated the feasibility and efficacy of MNs coated with FS and berberine for ocular applications. In the last 17 years, the research area of ocular MNs technology has advanced significantly. In this section, we provide a comprehensive overview of different PSMNs used in ophthalmology—MNPs, spring-loaded microneedles (SLMNs), and contact lens-shaped microneedles (CLSMNs)—classified based on the diverse therapeutic requirements of ocular treatments.

### Microneedle Patch Systems

MNPs have emerged as a pivotal innovation in drug delivery owing to their simple manufacturing process, cost-effectiveness, drug efficacy, and user-friendly operation. Considering the anatomical confines of the ocular sphere and the diverse requirements of drug delivery, the design parameters of MNPs are judiciously tailored to 200–1000 μm in length and 300–750 μm in height. Some common configurations of the MNP arrays are 3 × 3, 5 × 5, 10 × 10, and 20 × 20 arrays, which are customized based on the targeted application. MNPs designed for ocular administration can be classified into three principal categories: solid-coated, hollow, and dissolvable polymer MNPs. MN leverage nanotechnology to significantly enhance the efficiency and specificity of drug or therapeutic molecule delivery, thereby aiming to achieve superior therapeutic outcomes while minimizing side effects [309]. For instances, Huang et al. have developed a novel class of microneedles based on iron oxide with V–O–Fe coordination sites, known as spiky artificial peroxidase (V-Fe_2_O_3_), specifically designed to combat methicillin-resistant Staphylococcus aureus (MRSA) [310, 311]. Experimental investigations and theoretical computations have demonstrated that V-Fe_2_O_3_ microneedles can achieve a dual-functional “capture and kill” mechanism locally, attributed to their spiky morphology and the generation of a substantial amount of reactive oxygen species (ROS). This innovative approach leverages the unique physicochemical properties of V-Fe_2_O_3_ to enhance the antimicrobial efficacy against drug-resistant pathogens, offering a promising therapeutic strategy in the battle against MRSA infections. The integration of nanotechnology within MN systems allows for the precise manipulation of drug release profiles, ensuring targeted delivery to the affected areas. This targeted approach not only optimizes the therapeutic index by concentrating the drug at the site of action but also reduces systemic exposure, leading to a diminished incidence of adverse effects [312]. Consequently, MN technology, empowered by nanotechnology, holds great promise for advancing personalized medicine and improving patient care.

**Fig. 17:**
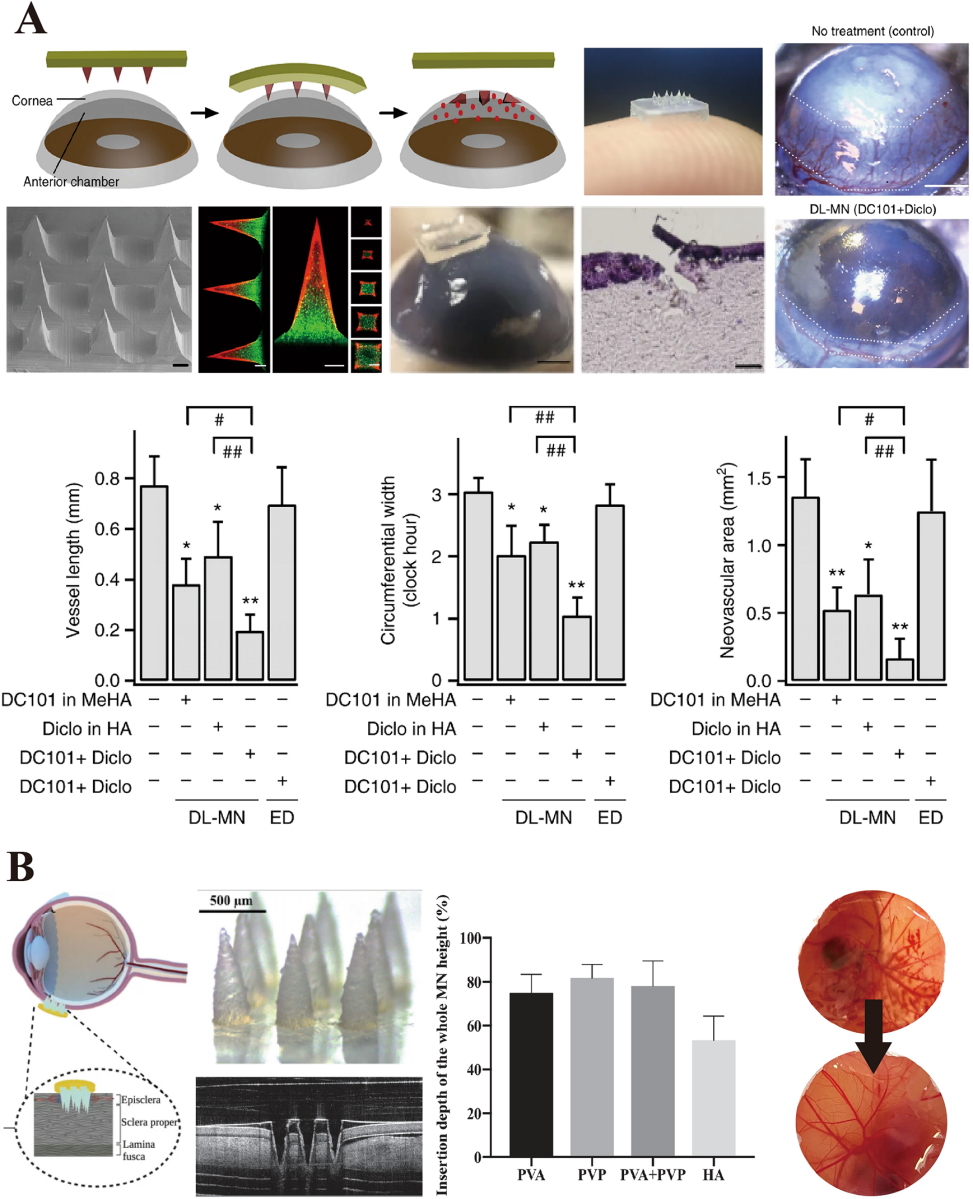
Application types of PSMNs in ophthalmology. **A** Autonomously implantable dual-layer micro-pharmaceutical reservoirs for the management of neovascular conditions. Reproduced under the terms of the Creative Commons CC BY license [50]. Copyright 2018, The Author(s), published by Springer Nature. **B** Swiftly disintegrating bilayered microneedles for the gentle and effective delivery of proteins, specifically targeted at remedying disorders of the retinal vasculature. Reproduced under the terms of the Creative Commons CC BY license [47]. Copyright 2022, The Author(s), published by Springer Nature

Solid-coated MNPs are engineered to perforate corneal or scleral surfaces, creating conduits that enable targeted drug transport to sites of pathology. MNPs are constructed from metals, Si, or glass and, hence, are non-degradable. Polysaccharide materials, including HA, CMC, sucrose, dextran, and CS, are typically used to coat the drug formulation on MNPs. Solid-coated MNPs are predominantly employed in the management of corneal (Fig. 17A) and scleral diseases such as corneal neovascularization, glaucoma, and posterior segment conditions (e.g., neovascular AMD) [47, 50]. These ocular structures present formidable barriers to conventional local drug delivery systems; solid-coated MNPs, acting as miniature drug reservoirs, adeptly penetrate these barriers to deliver therapeutics into the corneal stroma and scleral tissues. Jiang et al. [155] used FS as a model drug and confirmed the superior bioavailability of MNPs. Nevertheless, these MNPs have limitations such as restricted drug-loading capacity, single-use design, and less-than-optimal biocompatibility.

Single hollow microneedles are a nascent iteration of MNPs. These are typically composed of borosilicate micropipettes or biodegradable polymers and are adept at injecting nano- and micro-formulations directly through microchannels to target intraocular sites [313]. They are particularly suitable for posterior segment (sclera, choroid, and ciliary body) therapies. Notably, Patel et al. [204] employed hollow MNPs to administer nano- and microparticles to the suprachoroidal space of rabbit, pig, and human eyes and revealed the importance of the length of MNPs, applied pressure, and particle size for successful drug delivery. Hollow MNPs can deliver both macromolecular and small-molecule drugs at large doses, although their use may necessitate supplementary equipment to control pressure and dosing, and their disposability contributes to biohazardous waste generation.

**Fig. 18:**
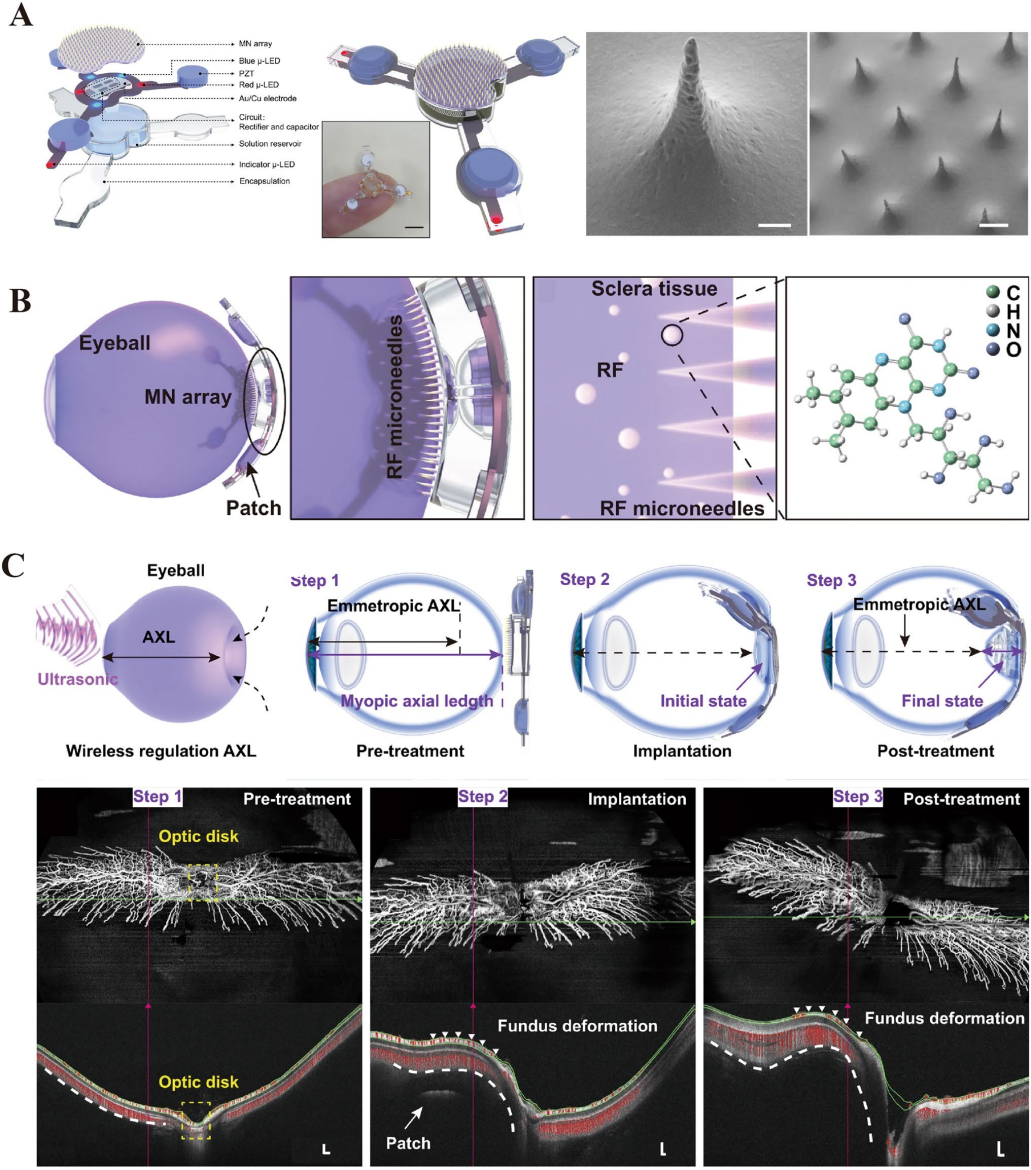
**A** Contact-free ocular modulation patch that operates without batteries or wired connections, designed to provide treatment for high myopia. **B** The working mechanism of this microneedles. **C** In vivo experiments of this microneedles. Reproduced under the terms of the Creative Commons CC BY license [315]. Copyright 2024, The Author(s), published by Springer Nature

Dissolvable polymer MNPs have attracted widespread interest, as they circumvent the limitations of hollow and solid-coated MNPs [158, 314]. These MNPs are fabricated from polysaccharides and biodegradable biocompatible polymers that exhibit rapid dissolution and high drug release efficacy. They have been applied to the treatment of both the anterior and posterior segments of the eye. Wu et al. [47] fabricated rapid-dissolving dual-layer MNPs using a matrix of HA with PVA and PVP, which dissolved within 150 s while preserving the bioactivity of the model protein OVA (Fig. 17B). As mentioned earlier, the integration of MNs and electrical signals is currently a popular topic, which is also true for the treatment of eye diseases. MNs have made significant progress in treating myopia. Zhong et al. [315] proposed a wireless battery-free eye modulation patch to correct high myopia and prevent recurrence (Fig. 18A). The patch consists of piezoelectric transducers, an electrochemical micro-actuator, a drug MN array, LEDs, a flexible circuit, and biocompatible encapsulation. The drug loaded into the MN array, which is composed of PVP and riboflavin, can be delivered to the posterior sclera through this system, and the entire system does not require batteries (Fig. 18B) [315]. In vivo experiments demonstrated that the patch successfully reduced the axial length of a rabbit eye by 1217 μm and increased the sclera strength by 387% (Fig. 18C) [315]. This study showed that the patch offers a promising avenue for the clinical treatment of high myopia.

MNPs have advantages such as cost efficiency, substantial drug-loading capacities, and controllable release rates, all of which are coupled with excellent biocompatibility. However, the solubility of their polysaccharide constituents poses a challenge to their mechanical integrity, a problem that future research should aim to resolve.

### Spring-Loaded Microneedles

**Fig. 19:**
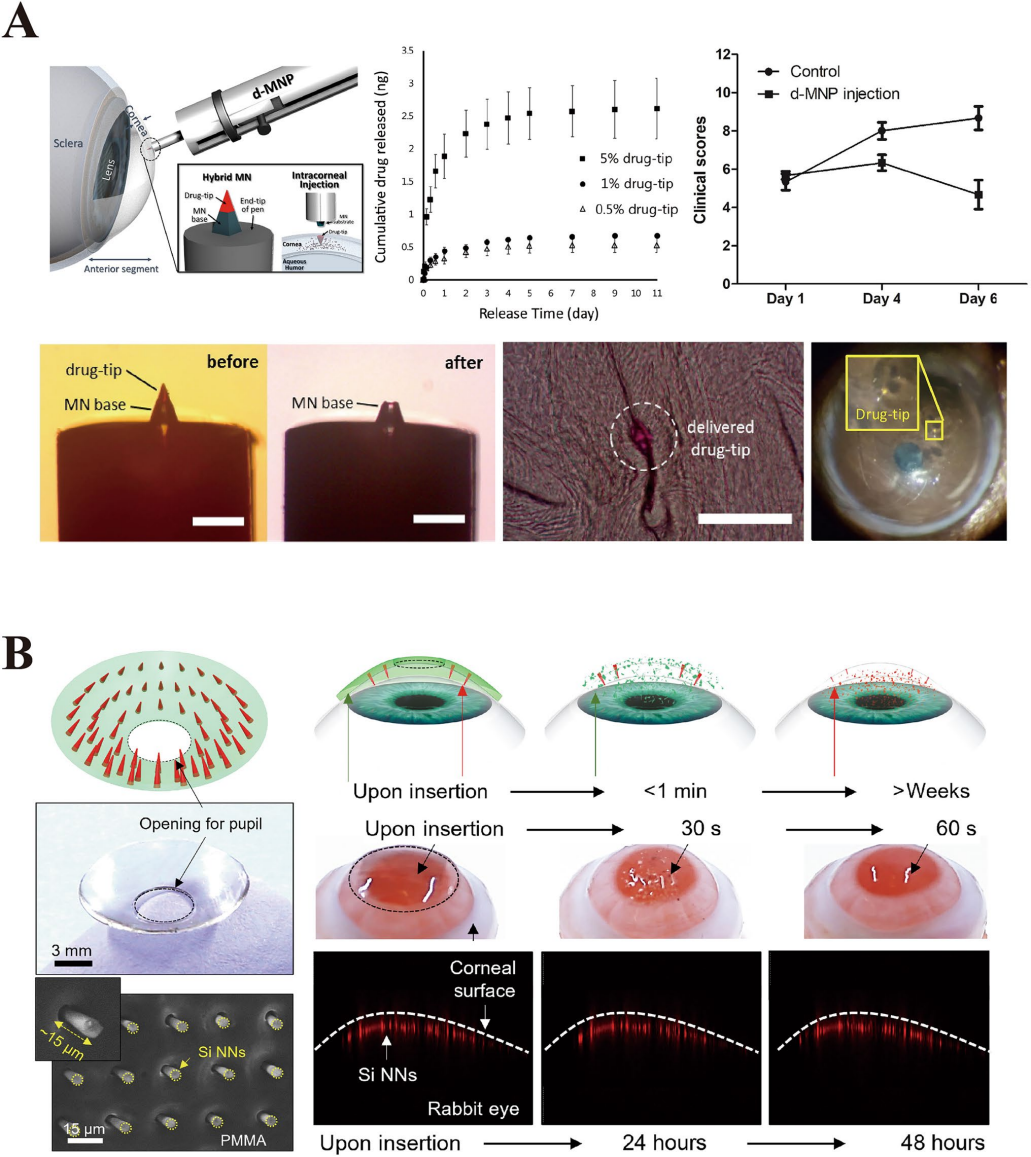
**A** Intracorneal implantation technique utilizing a removable hybrid microneedle to facilitate prolonged drug release. Reproduced with Permission [317]. Copyright 2018, Acta Materialia Inc. **B** Degradable silicon-based nanoneedles designed for the targeted delivery of medication to the eye for the treatment of corneal neovascularization. Reproduced under a Creative Commons Attribution NonCommercial License 4.0 (CC BY-NC) [319]. Copyright 2022, The Authors, published by Science Advances

SLMNs represent a novel paradigm for drug delivery devices that harness the mechanical force of a spring to insert MNs into the skin or other tissues. This device is particularly well-suited for treating posterior segment eye diseases, such as AMD, where traditional long-needle injections cannot control the penetration depth owing to the limited length of the posterior segment of the eye, which is a few centimeters deep. Inspired by the successful application of MNs in scleral delivery, Park et al. developed SLMNs. The SLMN device consists of a coated MN tip, a plunger, a trigger, a spring, and a slot [316]. This device leverages the spring energy to effectively deliver small-molecule drugs such as RhB into scleral tissues. Their SLMNs (a solution of RhB and sodium CMC onto resin tips) achieved a needle insertion depth of 512.42 ± 12.56 μm; after 6 h, the drug exhibited a vertical diffusion range of up to 1567.54 ± 360.12 μm and a horizontal diffusion range of up to 2740.83 ± 165.58 μm. Lee et al. [317] developed a detachable SLMN device that enables the insertion of degradable polymer needle tips into the eye through physical impact (Fig. 19A). By optimizing the needle tip size, the injection dwell time, and the insertion depth, they successfully delivered micro-sized, drug-loaded needle tips—approximately 1/100th the width of a mouse’s eye—into the soft and thin (> 150 μm thick) mouse cornea. The SLMN device was effectively inserted into the eye with an external force, providing sufficient penetration depth with minimal impact on vision, and shows potential as an alternative to eye drops for repeated intraocular administration [314, 318].

Despite the promising performance of SLMNs for ocular insertion, the limited number of needle tips used per insertion results in a relatively small drug delivery volume per application, necessitating multiple insertions to achieve therapeutic effects. Future studies should attempt to enhance the drug load per delivery and streamline the operational procedures to broaden the clinical application and convenience of SLMNs. Further investigation into the long-term safety and efficacy of SLMNs is essential to ensuring their reliability and practicality in ophthalmic treatments.

### Contact Lens-Shaped Microneedles

CLSMNs are a burgeoning innovation in ophthalmic therapy [320]. They combine the convenience of wearing contact lenses with the precision of MN drug delivery, offering a cutting-edge method for treating a range of ocular ailments. Researchers have developed CLSMNs that conform to the typical human corneal curvature range of 8.3–9.0 mm. Gade et al. [321] developed a CS-based contact lens and a dual-drug loading system using moxifloxacin (MF) hydrochloride and DM sodium phosphate. This CS-based contact lens exhibited superior mucoadhesiveness and antimicrobial efficiency compared with free drugs, indicating its efficacy in postoperative therapeutic scenarios.

Moreover, researchers have explored the integration of MNs with contact lenses to enhance drug delivery efficiency. Datta et al. [322] developed CLSMNs containing cyclosporine A (CsA) encapsulated within PVP MN tips. Drug distribution studies in porcine eyes demonstrated that the CsA concentrations in the cornea, aqueous humor, lens, vitreous humor, sclera, and choroid-retinal complex were 45.5%, 29.1%, 8.3%, 5.2%, 3.0%, and 0.2%, respectively, which proved the efficacy of the above-mentioned CLSMNs. Park et al. [319] developed a vertically ordered array of biodegradable Si MNs on a tear-soluble contact lens for enhanced needle sharpness and sustained drug release (Fig. 19B). They reported tear-soluble contact lenses with transmittance comparable to or superior to commercial soft contact lenses and thinner profiles (50–180 μm). The CLSMNs were coated with a PVA film to achieve biphasic drug release.

Hence, CLSMNs have extensive application prospects as ocular drug delivery systems. Future studies should aim to further refine the design and fabrication processes of CLSMNs to enhance drug bioavailability and therapeutic outcomes while ensuring patient comfort and safety. CLSMN technology is anticipated to become a significant tool in ophthalmic treatment, offering patients more convenient and effective therapeutic options.

## Regulation and Commercialization of PSMNs

As PSMNs are cutting-edge drug delivery systems, there should be a regulatory, evaluation, and commercialization framework crucial for ensuring patient safety, enhancing therapeutic outcomes, and achieving market success. At the regulatory level, national drug regulatory agencies, such as the FDA and the National Medical Products Administration (NMPA) of China, should establish strict guidelines and approval processes to ensure the safety and efficacy of PSMNs. These agencies aim to require extensive preclinical studies, animal testing, and human clinical trials to comprehensively assess the pharmacological effects, toxicological characteristics, and clinical efficacy of a product before it can be marketed. Additionally, the regulatory framework includes rigorous scrutiny of the manufacturing process, quality control, and product labeling to ensure product quality and consistency.

In terms of evaluation, the performance assessment of PSMNs is a critical step in the commercialization process. The assessment of drug delivery efficiency typically involves a systematic analysis of drug bioavailability, pharmacokinetic curves, and therapeutic effects. The biocompatibility of PSMNs focuses on their interaction with human tissue, including potential immune responses and inflammatory reactions. The physical and chemical stability of PSMNs during storage and transportation is tested using accelerated stability testing and long-term stability trials to predict the shelf life of the product and its optimal storage conditions to ensure its efficacy and safety within the specified period.

Commercialization strategies such as market demand assessment, strategic pricing, marketing strategies, and the establishment of sales channels are essential to the success of PSMNs. Intellectual property protection plays a vital role in the commercialization process, preventing competitors from imitating and ensuring that researchers receive a reasonable return on their investment. Furthermore, strategic partnerships with pharmaceutical companies, medical device manufacturers, and other relevant enterprises can accelerate market penetration and application of PSMNs.

Numerous challenges remain. Changes in regulatory policies may affect product development and market entry timelines. The high cost and complexity of clinical trials may hinder the participation of startups. Additionally, intense market competition demands the development of PSMNs with competitive advantages and innovative features. Technological innovation and market dynamics provide new opportunities for the development of PSMNs but also require researchers to continually adapt and innovate.

Overall, PSMNs are expected to play a significant role in drug delivery. With in-depth research and technological advancements, PSMNs may offer safer and more effective treatment options to improve patients’ quality of life. Regulatory agencies, researchers, and market shareholders must work together to ensure that the regulatory, evaluation, and commercialization frameworks support the development and application of this innovative technology. Through interdisciplinary collaboration, sustained research, and policy support, PSMNs can realize their full potential in the medical field.

## Outlook and Conclusion

Polysaccharide-based microneedles (PSMNs) are a pivotal advancement in ocular drug delivery, presenting a harmonious blend of biocompatibility, efficacy, and patient-centric design. The integration of PSMNs into therapeutic strategies for eye diseases heralds a new era in which the challenges imposed by ocular barriers are not only acknowledged but also effectively surmounted. This review systematically examined the principles, fabrication methods, and applications of PSMNs, revealing their potential to revolutionize the management of ocular diseases.

However, researchers and market shareholders must navigate a dynamic landscape of regulatory frameworks and commercialization pathways to bring the PSMNs technology from the laboratory to widespread clinical use. Interdisciplinary collaboration is paramount in refining PSMN technology to satisfy stringent safety profiles and manufacturing standards. Further research is required to optimize the mechanical properties and degradation rates of PSMNs to tailor the drug release profiles for diverse ocular conditions. Clinical trials that focus on patient outcomes and compliance are critical for validating the efficacy and safety of PSMNs and ensuring their practical applications.

Moreover, the integration of PSMNs with emerging technologies, such as smart drug delivery systems and biosensors, should be explored. Such synergies could enable real-time monitoring of drug delivery and ocular health, further personalizing patient care. Advancements in materials science and nanotechnology could enable finer control over needle design and drug release kinetics, broadening the therapeutic window and minimizing systemic exposure.

In conclusion, PSMNs are at the forefront of a transformative shift in ocular pharmacotherapy. By aligning drug delivery technologies with the intricate requirements of the eye, PSMNs pave the way for a future where vision impairment and blindness are no longer inevitabilities of disease progression but are conditions that can be effectively managed with precision and a patient-first approach. The continued evolution of PSMNs will depend on the confluence of innovative research, patient-centric design, and a regulatory environment conducive to swift yet safe advancements. A future in which the burden of ocular diseases is significantly reduced by the touch of microscopic needles looks promising.
